# Light‐Activatable Hyaluronic Acid‐Derivatives Releasing Nitric Oxide and Their Delivery in the Skin

**DOI:** 10.1002/adhm.202500589

**Published:** 2025-04-18

**Authors:** Giuseppe Longobardi, Cristina Parisi, Federica Sodano, Ovidio Catanzano, Anna Di Porzio, Antonio Randazzo, Salvatore Sortino, Fabiana Quaglia

**Affiliations:** ^1^ Department of Pharmacy University of Napoli Federico II Via Domenico Montesano 49 Napoli I‐80131 Italy; ^2^ PhotoChemLab Department of Drug and Health Sciences University of Catania Viale Andrea Doria 6 Catania I‐95125 Italy; ^3^ Institute for Polymers Composites and Biomaterials (IPCB)–CNR Via Campi Flegrei 34 Pozzuoli I‐80078 Italy

**Keywords:** hyaluronic acid, light, microemulsions, nitric oxide, skin

## Abstract

Hyaluronic acid (HA), renowned for its hydrating properties, penetrates deeper into the skin at a very low molecular weight, promoting cellular repair and reducing inflammation. Nitric oxide (NO), a gaseous mediator with a very relevant role in many physiological and pathophysiological processes, is expected to complement the functions of HA in the skin through its vasodilatory, anti‐inflammatory, and regenerative properties. Herein, a novel class of HA derivatives functionalized with NO photodonors (NOPD) for light‐activated therapeutic applications is introduced. The HA derivatives HA‐NOPD1 and HA‐NOPD2 demonstrated NO release under blue light activation. Biological assays on HaCaT keratinocytes revealed enhanced proliferation and migration under light stimuli, underscoring the therapeutic potential of HA‐NOPD1. The derivatives are formulated into microemulsions (MEs) to allow their skin transport. MEs loaded with the photoresponsive derivatives are stable in the dark and provide effective NO photorelease with higher quantum yields than the free compounds. Skin permeation studies using porcine and artificial membranes confirmed that HA‐NOPD2 distributed in all the skin layers, reaching the dermis and releasing NO in situ.

## Graphical abstract

This work explores the development of photoactivatable hyaluronic acid (HA) derivatives functionalized with nitric oxide (NO) photodonors for light‐controlled therapeutic applications. The HA derivatives demonstrate enhanced keratinocyte proliferation and migration, effective NO release, and skin penetration when formulated as microemulsions. These findings highlight the potential of HA and NO to treat dermatological disorders.

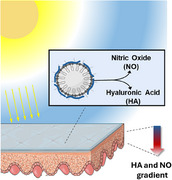

## 1 Introduction

Hyaluronic acid (HA) is a key player in maintaining skin integrity. Due to its ability to retain water, promote tissue repair, and interact with skin cells and the extracellular matrix, HA is essential for skin hydration, elasticity, and regeneration. The interaction between HA with specific molecular weight and cell surface receptors (CD44, RHAMM, TLR2, and TLR4, LYVE‐1) regulates cell behavior, including proliferation, migration, differentiation, and inflammation.^[^ ^1^ ^]^ From the strict chemical viewpoint, HA versatility stems from the abundance of hydroxyl and carboxylic acid groups, allowing straightforward modifications with various functional groups.^[^ ^2^ ^]^

Nitric oxide (NO) is a gaseous mediator relevant to various physiological and pathophysiological processes, usually stored in biological tissues as NO‐precursors.^[^ ^1^, ^3^ ^]^ These derivatives are particularly abundant in the skin, providing a protective barrier and an antimicrobial defense, establishing and maintaining circulation and melanogenesis, and promoting erythema in response to ultraviolet light exposure.^[^ ^4^ ^]^

Using NO for therapeutic purposes is challenging due to several factors related to its chemical properties, biological reactivity, and delivery requirements. Short half‐life (ca. 5 s) in biological systems makes NO act locally, confining its effects in the restricted region where it is generated. Since the biological effects of NO are strictly related to its amount, it is crucial to fine‐tune NO levels to leverage its therapeutic benefits while avoiding the risk of toxicity.^[^ ^5^ ^]^ The gaseous nature of NO makes its delivery into the body in a consistent and time‐controlled dose challenging.^[^ ^6^ ^]^ Several synthetic NO precursors that can store NO as part of their molecular or macromolecular skeleton and deliver it spontaneously have significantly increased over the last two decades.^[^ ^3^, ^7^ ^]^

It is expected that NO can complement the functions of HA in the skin through a variety of synergistic mechanisms, particularly in processes related to wound healing, tissue regeneration, inflammation modulation, and vascular function. Spontaneous NO releaser and high molecular weight HA delivered in an emulsion or a liposome formulation were found nontoxic to fibroblasts^[^ ^8^ ^]^ and useful to treat multimodal androgenetic‐alopecia therapy due to pro‐angiogenic properties.^[^ ^9^ ^]^ Furthermore, some examples of HA covalently conjugated with NONOates have been developed and employed as antimicrobial materials to treat infected wounds.^[^ ^10^ ^]^

The main limitation of spontaneous NO releasers resides in uncontrolled NO release, limited duration of action, difficulty in regulating the dose, off‐target effects, and lack of specificity. Furthermore, spontaneous NO‐releasing molecules are often chemically unstable and sensitive to environmental conditions (i.e., pH, temperature), leading to reduced shelf life and inconsistencies in therapeutic performance.

The peculiar features of the light make it the most elegant trigger to initiate the release of bioactive molecules with precise control in both space and time.^[^ ^11^ ^]^ The easy manipulation of light in terms of energy, intensity, location, and duration makes this trigger unique for accurate, non‐invasive regulation of the release processes without affecting the physiological values of pH and temperature of the living tissues. NO precursors activatable by light stimuli, namely NO photodonors (NOPDs), offer outstanding precision in regulating spatiotemporal NO release, avoiding suboptimal therapeutic levels.^[^ ^1^, ^12^ ^]^ NOPDs are based on the general concept of photocaging, where the NO molecule is covalently integrated within a photoresponsive chromogenic unit until the activation of the excitation light breaks the covalent bond and liberates the “caged” NO. NOPDs can be combined through robust covalent linking with other molecules to form molecular hybrids that share the same molecular skeleton and other functionalities, such as a targeting element, a fluorescent reporting function, or a second therapeutic species.^[^ ^1^, ^12^ ^]^

Despite their enormous potential, the exploration of NO‐releasing HA‐based nanocarriers under the input of light remains relatively limited in the literature, with only a few studies delving into this promising avenue of research.^[^ ^13^ ^]^

In this work, we report the development of two HA derivatives releasing NO under the input of environmental light to benefit from both HA and NO biological effects. These photoactivatable HA derivatives are formulated in a microemulsion for skin application to provide a proof‐of‐concept of their applicability for treating dermatological disorders.

## 2 Results and Discussion

### 2.1 Synthesis and Properties of the HA‐NOPD1 and HA‐NOPD2 Derivatives

**Figure 1:**
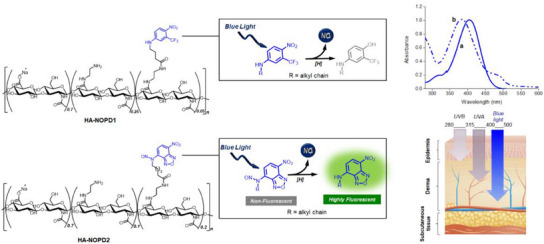
Molecular structures of **HA‐NOPD1** and **HA‐NOPD2** and, in the inset, the simplified NO photorelease mechanism of the NOPD motifs. Normalized absorption spectra of **HA‐NOPD1** a) and **HA‐NOPD2** b) in H_2_O:MeOH (1:1) and, at the bottom, the skin penetration capability of environmental light in the UV and blue visible region.

The HA derivatives **HA‐NOPD1** and **HA‐NOPD2** (**Figure** **1**) were synthesized according to the experimental procedures reported in SI. Briefly, HA was partially functionalized with amine terminations and then covalently linked with the carboxy‐terminated NOPDs based on nitroaniline and *N*‐nitroso amino nitrobenzofurazan chromophoric motifs developed in our group.^[^ ^14^ ^]^ The degree of functionalization with the NOPDs was ca. 16% and ca. 65% for **HA‐NOPD1** and **HA‐NOPD2**, respectively. The chromophoric motifs in NOPDs were demonstrated to work well after their covalent and non‐covalent integration in different polymeric scaffolds.^[^ ^15^ ^]^ This finding is not trivial since the environment of NOPDs, once integrated into the polymeric scaffold, can result in dramatic changes in photoresponsiveness. Both NOPDs release NO upon blue light excitation through a clean photochemical transformation, leading to two stable photoproducts (see insets of Figure 1). The nitroaniline derivative releases NO after intramolecular nitro‐to‐nitrite photorearrangement and, after H‐transfer, generates a phenol derivative, which is transparent in the visible region.^[^ ^11a^ ^]^ The *N*‐nitroso amino nitrobenzofurazan derivative releases NO by direct rupture of the N‐NO bond upon light excitation and, after H transfer, generates a highly green fluorescent product, which acts as an optical NO reporter useful for bio‐tracking.^[^ ^11b^ ^]^

The absorption properties of the NOPDs were well retained after their integration into the HA scaffold. The spectra shown in Figure 1 are very similar to those observed with the isolated chromophores, showing absorptions in the blue region with maxima at ca. 400 nm and tails extending up to ca. 500 nm. These spectral features are very suited for skin applications of the HA derivatives since blue light is currently employed to treat a variety of skin conditions^[^ ^16^ ^]^ penetrates epidermis/upper dermis up to 1 mm^[^ ^17^ ^]^ (see Figure 1), and can potentially activate local NO photorelease. Besides, the absorption of the HA derivatives in the range below 400 nm makes them an appropriate optical filter in the epidermal region against harmful UVA and UVB radiation.

**Figure 2:**
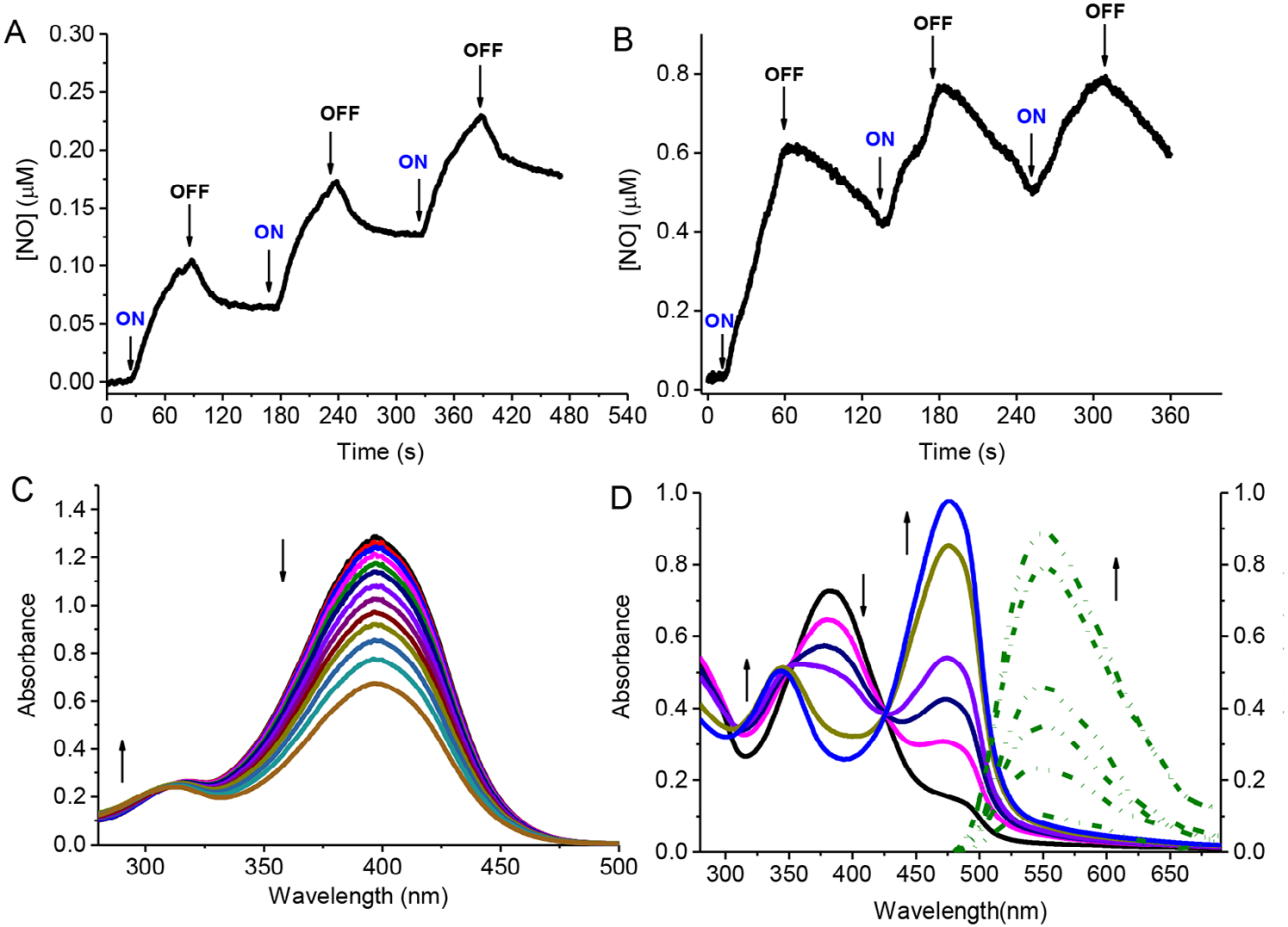
NO release profiles observed for air‐equilibrated solutions of **HA‐NOPD1** (150 µg mL^−1^) (A) and **HA‐NOPD2** (30 µg mL^−1^) (B) upon alternate cycles of light irradiation at λ_ac _= 405 nm. H_2_O: MeOH (1:1 v/v), T = 25 °C. (C) Absorption spectral changes observed upon exposure to the same solution as in (A) at λ_ac_ = 405 nm for time intervals from 0 to 56 min. (D) Absorption (solid lines) and fluorescence emission, λ_exc_ = 424 nm (dotted lines), spectral changes observed upon exposure of the same solution as in (B) at λ_ac _= 405 nm for time intervals from 0 to 13 min. The arrows in (C) and (D) indicate the course of the spectral profile with the illumination time.

**HA‐NOPD1** and **HA‐NOPD2** are stable in the dark and release NO under blue light activation (λ_ac_). This behavior is unambiguously confirmed by direct NO detection through an amperometric technique using an ultrasensitive NO electrode. **Figure** **2**A,B show a prompt NO release from both HA derivatives that instantaneously stops in the dark and restarts as the illumination source is turned on again.

Figure 2C,D illustrate the parallel photolysis profiles. In the case of **HA‐NOPD1**, the irradiation leads to a significant bleaching of the main absorption band of the NOPD component at ca. 400 nm without change in the maximum position. Irradiation of **HA‐NOPD2** shows the bleaching of the main absorption band at ca. 382 nm and the formation of a new, intense absorption at 474 nm accompanied by clear isosbestic points. Besides, the evolution of the fluorescence emission spectra observed upon irradiation of **HA‐NOPD2** shows that the photodecomposition leads to a dramatic increase of the green emission with λ_max_ = 550 nm and a fluorescence decay exhibiting a dominant component (ca. 80%) with a lifetime of 2.4 ns (Figure S7, Supporting Information). These changes in the overall spectral features are in excellent agreement with those observed for the isolated NOPDs^[^ ^14^ ^]^ and confirm in both cases that their integration into the HA polymeric scaffold does not lead to any additional photochemical pathways competitive with the NO photorelease. The values of the quantum yields for the NO photogeneration, Φ_NO_, which were (3.0 ± 0.2)x10^−3^ and 0.16 ± 0.02 for **HA‐NOPD1** and **HA‐NOPD2**, respectively, are also in excellent agreement with those already reported for the isolated NOPDs under similar experimental conditions.^[^ ^14^, ^15^ ^]^

### 2.2 Biological Activity on HaCaT Cell Proliferation and Migration

**Figure 3:**
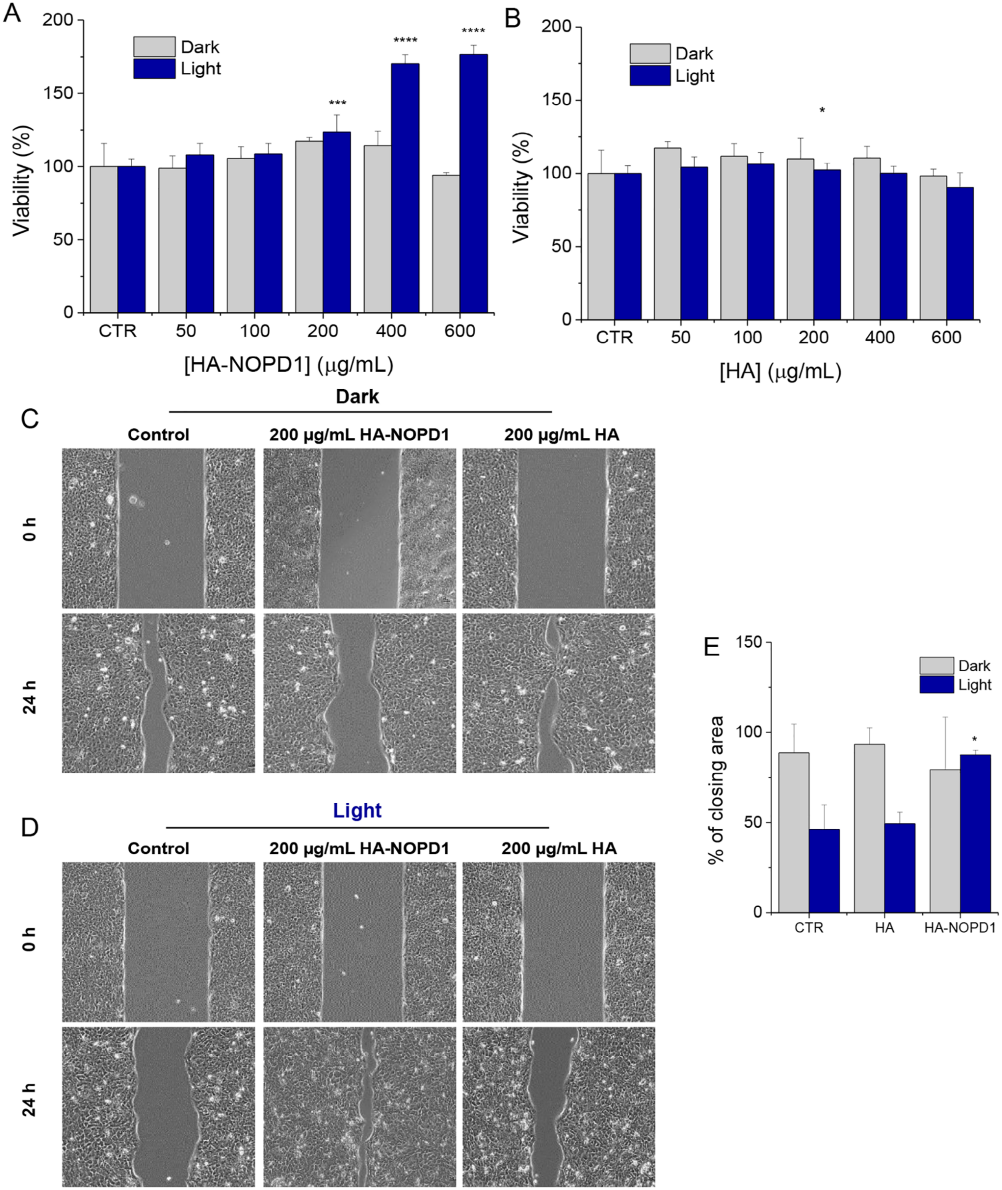
MTT cytotoxicity profiles resulting from the 24‐h treatment of HaCaT cells with increasing concentrations of (A) **HA‐NOPD1** or (B) HA, under dark and light conditions. Representative light microscope images are reported in Figure S8 (Supporting Information). (C) Representative light microscope images of the in vitro wound healing assay conducted on HaCaT cells in the presence of 200 µg mL^−1^ **HA‐NOPD1** or HA, under dark conditions or ((D) after 15 min of irradiation. A blue led was employed to irradiate cells (21.9 mW cm^−2^ at 420 nm). Cell migration was assessed at 0 and 24 h post‐scratch and quantified (E). Scale bar: 50 µm. Histograms show the mean ± SD of two independent experiments. The statistical significance of values *vs* CTR was calculated using a one‐way ANOVA test on GraphPad Prism 10 (*: *p *< 0.05; ***: *p *< 0.001; ****: *p *< 0.0001).

The effects of **HA‐NOPD1** on human keratinocyte proliferation were preliminarily assessed by exposing HaCaT cells^[^ ^18^ ^]^ to increasing concentrations of the HA‐derivative (50–600 µg mL^−1^). Cell irradiation was carried out at a dose of ca. 20 J/cm^2^, which should not alter cell metabolic activity.^[^ ^19^ ^]^ This light dose allowed **HA‐NOPD1** to absorb photons and resulted in limited light‐induced cytotoxicity (<40%). Intriguingly, **HA‐NOPD1** boosted HaCaT cell proliferation under blue light activation by ≈20%, 70%, and 75% at 200, 400, and 600 µg mL^−1^ concentrations, respectively (**Figure** **3**A). In contrast, no significant changes in cellular proliferation were detected when conducting the same treatments in the dark, where NO is not generated (Figure 3A). These findings suggest that the photoresponsive unit of **HA‐NOPD1** could be responsible for the observed increase in keratinocyte growth. To further explore this hypothesis, HaCaT cells were exposed to increasing concentrations of unmodified HA ranging from 50 to 600 µg mL^−1^ (as for **HA‐NOPD1**) under light and dark conditions. Unmodified HA (lacking the NOPD unit) did not appreciably affect the percentage of cell growth, regardless of light exposure (Figure 3B), in line with previous reports.^[^ ^20^ ^]^ Overall, these results indicate that the enhanced proliferation of human keratinocytes found after treatment with concentrations > 200 µg mL^−1^ of **HA‐NOPD1** can be ascribed to light‐triggered NO release in an appropriate amount.

Considering that NO is expected to complement the functions of HA in the skin synergistically, the ability of **HA‐NOPD1** to promote HaCaT cell migration in vitro was investigated using the standard wound healing assay. The concentration of **HA‐NOPD1** chosen for these experiments was 200 µg mL^−1^, as it was the lowest concentration that could significantly stimulate HaCaT proliferation. As shown in Figure 3C–E, the migration of cultured HaCaT cells was significantly enhanced by exposure to **HA‐NOPD1** exclusively under light conditions. On the other hand, treatment with HA (at the same concentration) had no relevant effects on HaCaT cell migration, neither in the light nor in the dark. Altogether, the results of the biological experiments demonstrate that **HA‐NOPD1** can stimulate both keratinocyte proliferation and migration, suggesting its promising potential as a therapeutic agent for dermatological disorders.

### 2.3 Development of a ME for Skin Delivery of the HA Derivatives

**Figure 4:**
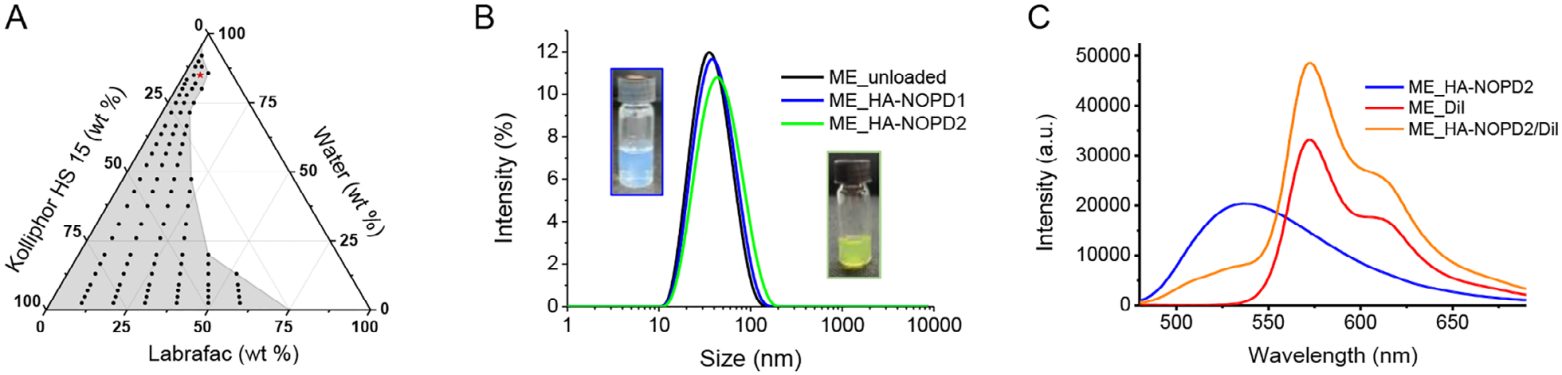
(A) Pseudo‐ternary diagram prepared for various surfactant (S) oil (O) and water (W) ratios. The red dot denotes the specific composition (10% Kolliphor HS 15, 5% Labrafac™ Lipophile WL1349, and 85% water) of the chosen formulation. (B) Size distribution of the microemulsion unloaded or loaded with **HA‐NOPD1** (ME_**HA‐NOPD1**) or **HA‐NOPD2** (ME_**HA‐NOPD** **2**). ((C) Fluorescence emission spectra of ME_**HA‐PD2** [25 µg mL^−1^], ME_DiI [25 µg ml^−1^] and ME_**HA‐PD2**/DiI [25 µg mL^−1^] (λ_ex_=470 nm).

HA functionalized with NOPDs is not well soluble in neat water and needs to be formulated to encourage skin penetration. To this purpose, we focused on MEs, which are widely employed for the skin delivery of pharmaceuticals and cosmetic ingredients^[^ ^21^ ^]^ and produced by green methods, avoiding organic solvents, heat, and high‐speed mixing. The components of an ME function as penetration enhancers when applied topically by temporarily reducing the skin barrier effect and facilitating drug transport through the skin.^[^ ^22^ ^]^ The system composition and the physicochemical characteristics of the components determine the spontaneous emulsion process.^[^ ^23^ ^]^ MEs typically consist of at least three ingredients: an aqueous component, an oily component, and an emulsifying agent. Generally, non‐ionic surfactants with a hydrophilic/lipophilic balance (HLB) between 12–18 are preferentially used in MEs as they can rapidly diffuse from the oil to the aqueous phase and provide a macroscopically homogenous and thermodynamically stable isotropic system. In this work, Kolliphor HS 15 was selected as the surfactant as it possesses an HLB in the range 14–16 and contains a PEG chain in the structure, providing ME with stability and protein repulsion capacity. Kolliphor HS 15 was mixed in different weight ratios with Labrafac™ Lipophile WL1349 as the oil phase and added with water to construct the pseudo‐ternary diagrams necessary to identify the area of spontaneous ME formation (**Figure** **4**A). The grey region of the phase diagram includes conditions where stable MEs are generated, whereas the remaining portion refers to lactescent emulsions. The results consistently indicate that the thermodynamic stability of the ME is preserved even at high volumes of the aqueous phase and minimal volumes of the oil phase. Based on the pseudo‐ternary diagram, we screened the properties of a large panel of compositions (Table S1, Supporting Information), all within the ME formation area, trying to keep the surfactant concentration as low as possible to limit toxicity. We selected the most suitable ME to deliver HA derivatives according to the colloidal properties (Table S1, Supporting Information). Transparency, size <100 nm, and polydispersity index ≤ 0.2 were the applied selection criteria to refine the number of formulations. Finally, a short‐term stability test was conducted, and a single formulation meeting all the stability criteria was selected and loaded with HA derivatives. The red dot in Figure 4A reports its specific composition (10% Kolliphor HS 15, 5% Labrafac™ Lipophile WL1349, and 85% water).

The MEs loading **HA‐NOPD1** or **HA‐NOPD2** show a narrow size distribution (Figure 4B) and an average hydrodynamic diameter (D_H_) of ≈40 nm with a PDI of ≈0.2. No difference in D_H_ and PDI is observed compared to the unloaded MEs. Similarly, the value of zeta potential remains around neutrality (−0.9 ± 0.3 mV for ME_un, −0.5 ± 0.2 mV for ME_**HA‐NOPD1**, and −0.4 ± 0.2 mV for ME_**HA‐NOPD2**) as well as the pH, which is ca. 6 in all the cases.

The arrangement of HA in the MEs was investigated by Förster Resonance Energy Transfer (FRET) analysis (Figure 4C). For this experiment, we employed **HA‐PD2**, the fluorescent product formed after NO loss by **HA‐NOPD2**, as an energy donor (see Supporting Information) and DiI as an energy acceptor. **HA‐PD2** and DiI were integrated into the ME as a single molecule (ME_**HA‐PD2** and ME‐DiI) or in combination (ME_**HA‐PD2**/DiI). FRET is expected to occur only if **HA‐PD2** (added in the aqueous phase of the ME) and DiI (added in the oily core of the ME) are in close proximity. The colloidal properties of the MEs loaded with **HA‐PD2** and DiI do not differ from those of the MEs containing the single components (Table S2, Supporting Information). The absorption and emission spectra of control MEs loaded with single components are reported in Figure S9 (Supporting Information). As illustrated in Figure 4C, the emission spectrum of ME_**HA‐PD2**/DiI excited at 470 nm (isosbestic point) shows the suppression of **HA‐PD2** emission and the concurrent enhancement of DiI emission. The occurrence of FRET between the **HA‐PD2** and DiI suggests that **HA‐PD2**, due to its amphiphilic nature, is located at the interface between the oily and the water phase, with the lipophilic photocage facing the oily phase and the HA chain toward the aqueous environment.

**Figure 5:**
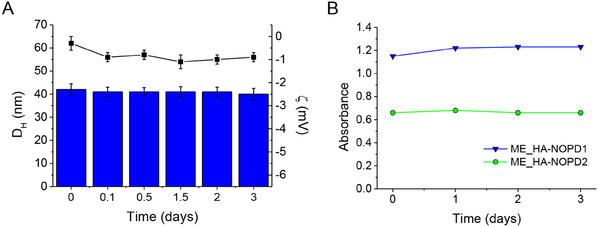
(A) Average size (bars) and zeta potential (symbols) of ME_**HA‐NOPD2** during time evaluated in the dark at room temperature. (B) Absorbance of ME_**HA‐NOPD1** (150 µg mL^−1^) and ME_**HA‐NOPD2 (**30 µg mL^−1^) at absorption maxima 404 and 384 nm, respectively, stored in the dark at room temperature.

ME_**HA‐NOPD1** and ME_**HA‐NOPD2** remained completely transparent during storage, and no macroscopic changes were observed. Furthermore, no phase separation occurred in any formulation, visually appearing as a homogeneous phase without precipitates or lumps. As a representative trend, the D_H_ and zeta potential (ζ) of ME_**HA‐NOPD2** at room temperature over time are reported in **Figure** **5**A. The stability of the ME_**HA‐NOPD1** and ME_**HA‐NOPD2** was assessed by collecting the absorption spectra at room temperature over 3 days (Figure 5B). Remarkably, both MEs showed no significant absorbance changes, according to no loss of NO under the tested experimental conditions.

**Figure 6:**
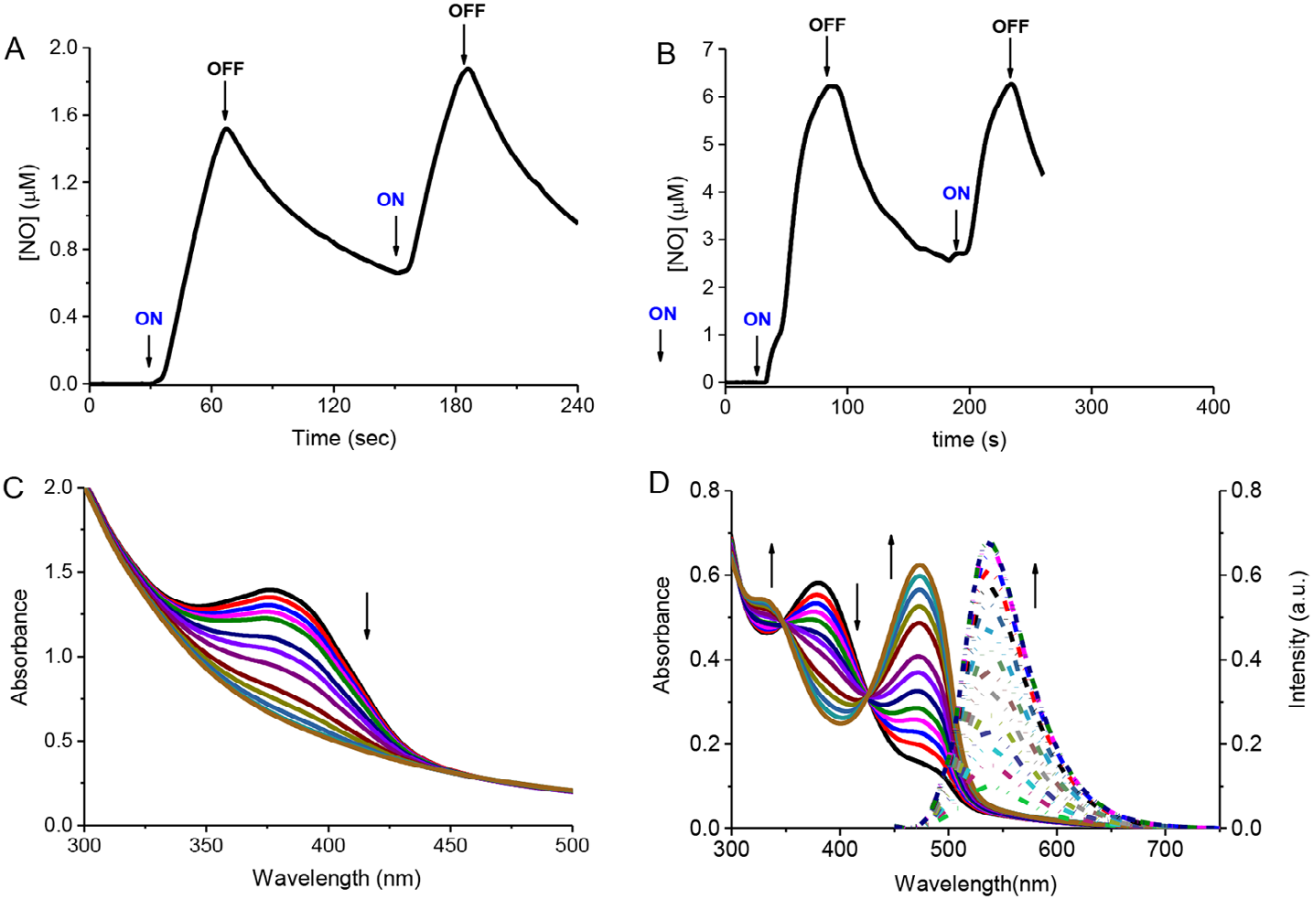
NO release profiles observed for air‐equilibrated ME of **HA‐NOPD1** (150 µg mL^−1^) (A) and **HA‐NOPD2** (30 µg mL^−1^) (B) upon alternate cycles of light irradiation at λ_ac_ = 405 nm. T = 25 °C. (C) Absorption spectral changes observed upon exposure of the same ME as in (A) at λ_ac_ = 405 nm for time intervals from 0 to 35 min. (D) Absorption (solid lines) and fluorescence emission, λ_exc _= 424 nm (dotted lines) spectral changes observed upon exposure to the same solution as in (B) at λ_ac _= 405 nm for time intervals from 0 to 7 min. The arrows in (C) and (D) indicate the course of the spectral profile with the illumination time.

As shown in **Figure** **6**A,B, both the HA derivatives release NO in a way exclusively controlled by visible light. Besides, apart from a shift of the absorption spectra toward lower wavelength, reasonably attributable to a solvent effect due to the specific microenvironment of the ME, the spectral profiles observed upon light irradiation (Figure 6C,D) were the same as those reported in the absence of ME (see Figure 2C,D, for the sake of comparison). Interestingly, loading HA derivatives in the ME affected the value of Φ_NO_, which increased up to (17 ± 0.2) x10^−3^ and 0.27 ± 0.03 in the case of **HA‐NOPD1** and **HA‐NOPD2**, respectively. This enhancement is not uncommon for these NOPDs when integrated within different surfactant‐based hosts ^[^ ^14^, ^24^ ^]^ and can be due to the active role of surfactant of the ME in providing efficiently abstractable hydrogens close to the phenoxy and anilinyl radical intermediates involved in the mechanism of the NO photorelease.

### 2.4 Skin Transport of HA Derivative

**Figure 7:**
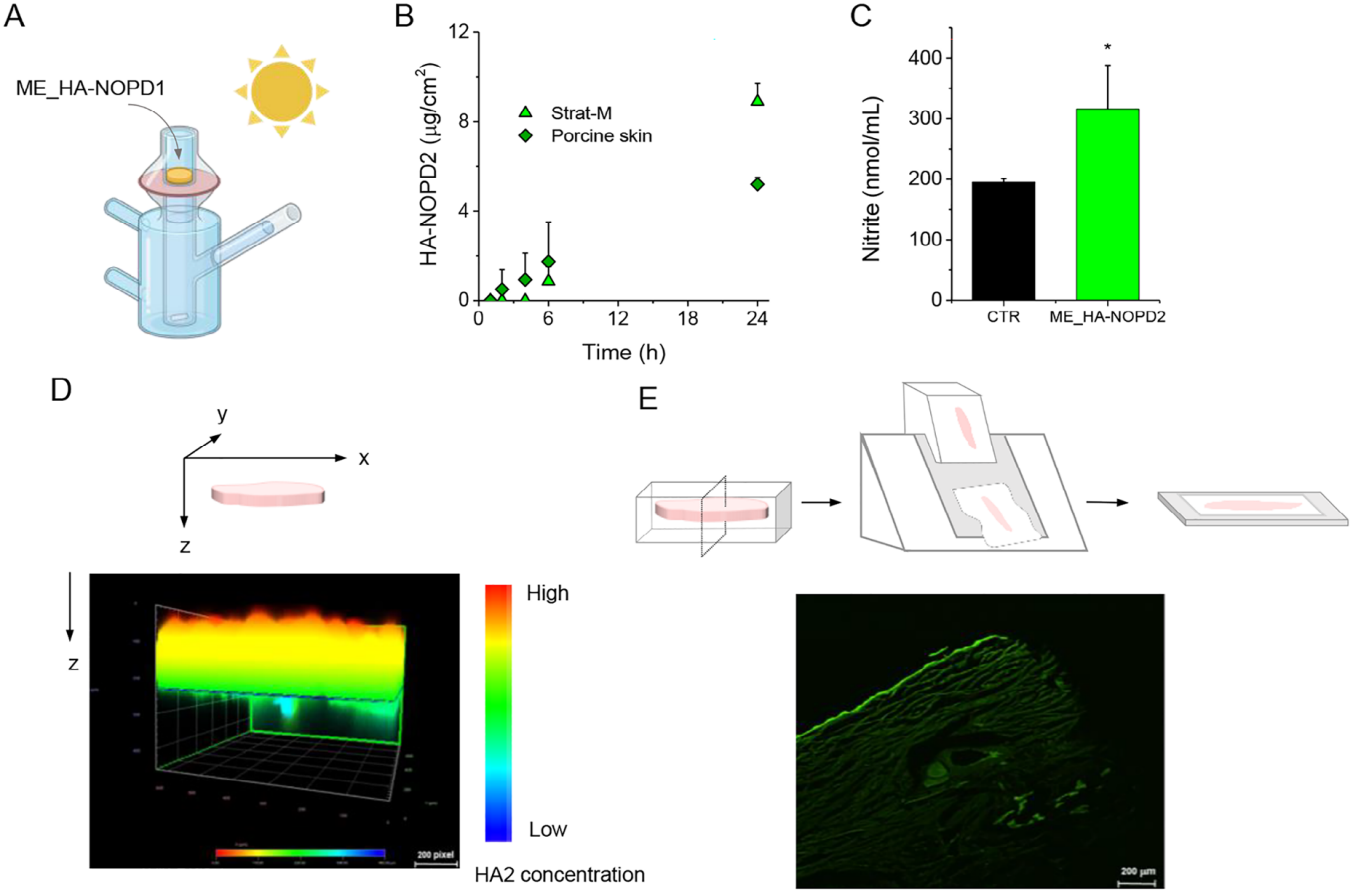
Permeation experiment on ME_**HA‐NOPD2** under environmental light. (A) ME_**HA‐NOPD2** was placed in the donor compartment whereas PBS pH 7.4 with 20% polysorbate 80 was poured in the receptor compartment. (B) Amount of **HA‐PD2** formed in the receptor compartment after NO photorelease by ME_**HA‐NOPD2** permeated through Strat‐M and porcine skin. Results are shown as mean ± the SD of three independent experiments. (C) Nitrite amount photogenerated in the receptor compartment after 24 h of ME_**HA‐NOPD2** permeation through porcine skin. A control experiment was run by adding PBS in the donor compartment, which is representative of the spontaneous production of NO in the skin (*P<0.01). Results are shown as mean ± the SD of three independent experiments. (D) Image of the z‐skin section and (E) Intensity gradient through z‐axis showing the distribution of the green fluorescent **HA‐PD2** derivative in the porcine skin after 24 h of permeation.

We selected **HA‐NOPD2** for skin transport studies since it produces the green, fluorescent reporter **HA‐PD2**, allowing tracking of the HA derivative and NO photorelease process in the skin. The permeation of **HA‐NOPD2** delivered via the ME through the skin was assessed in a typical transport experiment in vertical diffusion cells using both biological and artificial membranes. Live full‐thickness porcine skin was selected since its similarities with human skin in terms of thickness of the stratum corneum (SC), epidermis, dermis, and structure of hair follicles (both associated with sebaceous glands) make it the most simple viable tissue model in permeation studies^[^ ^25^ ^]^ and a reliable alternative to animal studies.^[^ ^26^ ^]^ We also tested skin transport through Strat‐M, an artificial membrane considered an alternative to avoid *ex vivo* live tissues and designed to provide comparable characteristics to human skin in permeating low molecular weight substances.^[^ ^27^ ^]^ Its architecture, comprising a compact upper layer coated with a lipid mixture that mimics the lipid component of the human SC and a porous lower layer simulating the layers of the epidermis and dermis, is engineered to mimic key structural and chemical features of human skin.^[^ ^28^ ^]^ The permeation experiment was conducted for 24 h. As shown in **Figure** **7**A, ME_**HA‐NOPD2** was placed in the donor compartment of the diffusion cells under environmental light.

As shown in Figure 7B, the amount of the **HA‐PD2** reporter in the donor compartment progressively increases over time, reaching ≈5 µg cm^−2^ (10% of the initial amount of **HA‐NOPD2**) after 24 h. **HA‐NOPD2** starts to permeate after a lag time of 6 h on the Strat‐M membrane, reaching ≈9 µg cm^−2^ (17%) after 24 h, a value much higher than that observed on porcine skin. In parallel, the amount of nitrites, the stable product of NO oxidation, was measured in the receptor compartment after 24 h (Figure 7C). A noticeable amount of nitrites was found when skin samples were treated with PBS (CTR) due to endogenous NO production through various mechanisms, including enzyme‐independent pathways.^[^ ^29^ ^]^ After 24 h of skin treatment with ME_**HA‐NOPD2,** a significantly higher nitrite generation than the CTR samples was found in the receptor compartment, which is reasonably due to NO photogeneration. The skin tissue was analyzed at the end of the transport experiment (24 h) by confocal microscopy to evaluate if there was any green fluorescence due to the reporter **HA‐PD2**. The analysis of skin cross‐section (Figure 7D) confirms the presence of the HA derivative in the epidermis and is in line with its ability to cross the skin and reach the donor compartment. We also imaged the whole tissue (z‐axes) and observed an intensity gradient of green fluorescence (Figure 7E) attributable to an **HA‐PD2** concentration gradient. **HA‐PD2** amount is the highest in the SC (red) and decreases progressively, moving toward the deep layers of the skin (blue). Since the SC in porcine ear skin is ≈21 µm thick, whereas the epidermis is ≈100 µm,^[^ ^25^ ^]^ these data demonstrate that **HA‐PD2** can reach the dermis.

**Figure 8:**
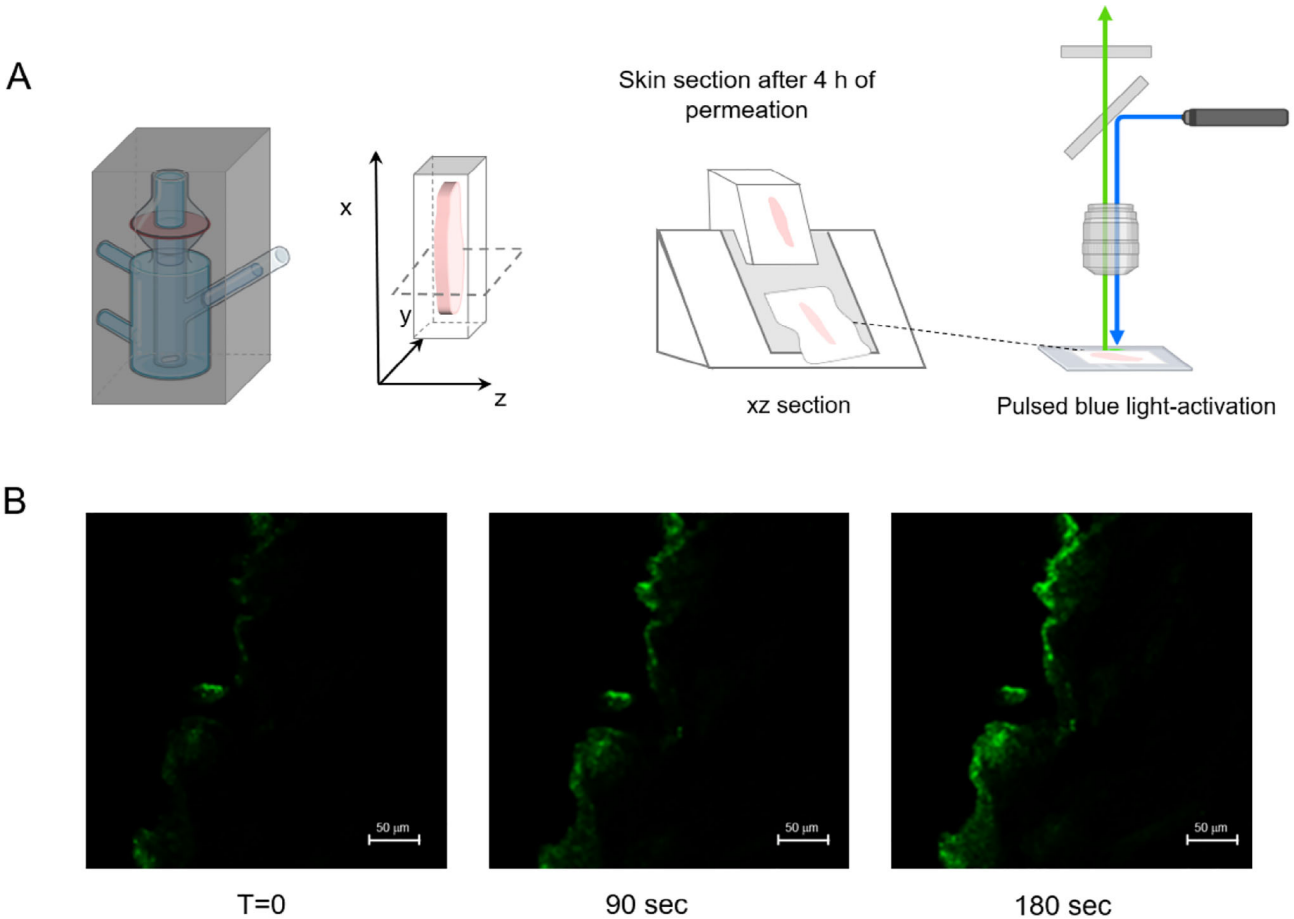
Permeation experiment on ME_**HA‐NOPD2** in the dark through porcine skin. (A) Set‐up of the experiment to demonstrate NO photorelease in the skin. The permeation was carried in the dark by pouring ME_**HA‐NOPD2** in the donor compartment and analyzing the tissue after 4 h. (B) xz‐ porcine skin section treated with blue light pulses (λ_ex_ = 488 nm; 2 µs) for 0, 90, and 180 s. The increase in green fluorescence is due to the photoconversion of the poorly emitting **HA‐NOPD2** into the highly emitting **HA‐PD2** upon NO decaging.

We carried out the same permeation experiment in the dark to demonstrate that NO is released in the skin under light input. After 4 h, the porcine skin was removed, washed, cross‐sectioned, and mounted on a glass slide (**Figure** **8**A). All these operations were carried out in the dark to avoid **HA‐NOPD2** photolysis. The skin membrane was placed under the confocal microscope and immediately observed (Figure 8B, left panel). The same sample was treated with laser‐generated pulses of blue light (λ_exc _= 488 nm) at intervals of 2 µs to stimulate NO release. The increase of the green fluorescence in the tissue due to the conversion of the low emitting **HA‐NOPD2** to the highly fluorescent **HA‐PD2** (Figure 8B, middle and right panels) unambiguously demonstrates that NO release is occurring in the skin.

Overall, these experiments unequivocally show that ME_**HA‐NOPD2** allows the HA derivative accumulation in the skin and the in situ release of NO under the input of light.

The ME allows the transport of the HA derivative into the deeper layers of the skin and offers significant advantages for several applications. NO can orchestrate the immune response and inflammation, induce vasodilation,^[^ ^30^ ^]^ providing a dynamic response to various pathogenic conditions. Moreover, NO can act in concert with oligo‐HA in increasing angiogenesis, collagen deposition, and endothelial cell proliferation.^[^ ^31^ ^]^ HA with a low and very low MW has proven to be capable of affecting the expression of numerous genes, including those involved in the differentiation of keratinocytes and the formation of intercellular tight junction complexes.^[^ ^32^ ^]^ More importantly, these HAs inhibit TLR3‐dependent inflammation through interaction with TLR4,^[^ ^33^ ^]^ weakening the pro‐inflammatory effects of the DAMP/TLR4 pathway. This suggests that HA may have the ability to reduce other TLR4‐related inflammatory processes, such as dermatitis or LPS‐induced inflammation.^[^ ^34^ ^]^ Besides, taking advantage of the synergistic action of NO and HA and the possibility of controlling the level of action of **HA‐NOPD1** and **HA‐NOPD2** in the skin through an appropriate delivery platform, the HA/NO win‐win strategy can also be exploited in cosmetic applications for rejuvenation and to promote hair growth. A patent application has been filed for this technology.^[^ ^35^ ^]^

## 3 Conclusion

In this study, we propose a novel strategy to combine the beneficial properties of HA and NO in skin disorders. HA derivatives were successfully synthesized by linking HA with different NO‐photoreleasing units, which preserved their excellent photochemical properties in the bioconjugate. HA derivatives even improved NO photoreleasing performance when formulated as MEs. We found a light‐dependent enhancement of keratinocyte proliferation and migration of HA derivatives, suggesting the potential synergistic role of HA and NO in promoting tissue repair and regeneration. Besides, an HA derivative forming a NO‐fluorescent reporting unit allowed a demonstration of NO release up to the deep skin layers under the input of environmental light. By combining the biological benefits of HA and the precision of light‐triggered NO release with nanotechnology, this work lays the foundation for advanced light‐activated therapeutic strategies in dermatology and beyond. Finally, we cannot exclude using blue LED in conjunction with HA‐NOPD MEs since blue light alone is currently employed to treat a variety of skin conditions.

## Conflict of Interest

The authors declare no conflict of interest.

## Supporting information



Supporting Information

## Data Availability

The data that support the findings of this study are available from the corresponding author upon reasonable request.
