# Supplementary material for: Light‐Activatable Hyaluronic Acid‐Derivatives Releasing Nitric Oxide and Their Delivery in the Skin
Source: Adv Healthc Mater. 2025 Apr 18;14(15):2500589. doi: 10.1002/adhm.202500589 (PMC12147995; doi:10.1002/adhm.202500589)
Supplement: Supplementary file 1 — Supporting Information [file ADHM-14-0-s001.docx]

Supporting Information

Light-Activatable Hyaluronic Acid-Derivatives Releasing Nitric Oxide and Their Delivery in the Skin

Giuseppe Longobardi,^†^ Cristina Parisi,^†^ Federica Sodano, Ovidio Catanzano, Anna Di Porzio, Antonio Randazzo, Salvatore Sortino,^*^ Fabiana Quaglia^*^

G. Longobardi, F. Sodano, A. Di Porzio, A. Randazzo F. Quaglia

Department of Pharmacy, University of Napoli Federico II

Via Domenico Montesano 49, I-80131 Napoli, Italy

C. Parisi, S. Sortino

PhotoChemLab, Department of Drug and Health Sciences, University of Catania

Viale Andrea Doria 6, I-95125 Catania, Italy

O. Catanzano

Institute for Polymers, Composites and Biomaterials (IPCB) – CNR

Via Campi Flegrei, 34, I-80078 Pozzuoli (NA), Italy

^*^E-mail: [quaglia@unina.it](mailto:quaglia@unina.it); [ssortino@unict.it](mailto:ssortino@unict.it)

^†^Equally contributed

**Table of Contents**

• Materials 3

• Instrumentation 3

• Synthesis 4

• Experimental Procedures 9

• References 14

• Table S1 15

• Table S2 16

• Figure S1 16

• Figure S2 17

• Figure S3 17

• Figure S4 18

• Figure S5 18

• Figure S6 19

• Figure S7 19

• Figure S8 20

• Figure S9 21

**Materials**

Caprylic/Capric Triglyceride (Labrafac^TM^ Lipophile WL1349) was kindly donated by Gattefossé (Saint-Priest, France). Macrogol (15)-hydroxystearate (Kolliphor^®^ HS 15) was purchased from BASF (Ludwigshafen am Rhein, Germany). Strat-M^®^ Membranes, PBS tablets, Tween^®^ 80, Nitrite Assay Kit (Griess Reagent), DMEM without phenol red and Mitomycin-C were obtained by Merck (St. Louis, MO, USA). L-Glutamine and 1,1′-Dioctadecyl 3,3,3′,3′tetramethylindocarbocyanine (DiI) perchlorate were purchased from Thermo Fisher Scientific (Waltham, MA, USA). Dulbecco’s Modified Eagle’s Medium (DMEM), Dulbecco’s Phosphate Buffered Saline (PBS), Fetal Bovine Serum (FBS), penicillin, streptomycin, and cell culture plasticware were provided by Euroclone S.p.a. (Milan, Italy). Dimethyl sulfoxide (DMSO) was supplied by HiMedia (Modautal, Germany). The CellTiter 96^®^ AQueous One Solution (Cell Proliferation Assay) was purchased from Promega (Madison, WI, USA). 2-well silicone culture-inserts in 35 mm µ-dishes were provided by Ibidi (Munich, Germany). Sodium hyaluronate (8-15 kDa) (HA) was purchased from Contipro a.s. (Dolní Dobrouč, Czech Republic) If not specified differently, reagents were obtained from Sigma Chemical Co. The water used for this study was purified and filtered using a Milli Q filter.

All reactions involving air-sensitive reagents were performed under nitrogen in oven-dried glassware using the syringe-septum cap technique. All solvents were purified and degassed before use.

Chromatographic separation was performed under pressure on Merck silica gel 60 using flash-column techniques. Reactions were monitored using thin-layer chromatography (TLC) carried out on 0.25 mm silica-gel-coated aluminum plates (60 Merck F_254_) and using UV light (254 nm) as visualizing agent. Unless otherwise specified, all reagents were used as received without further purification. Dichloromethane was dried over P_2_O_5_ and freshly distilled under nitrogen before use. DMF was stored over 3Å molecular sieves.

**Instrumentation**

^1^H and ^13^C NMR spectra were recorded on a Varian Unit Inova at 500 MHz. Chemical shifts (δ) are given in parts per million (ppm) and the coupling constants (J) in Hertz (Hz). The following abbreviations were used to designate the multiplicities: d = doublet, t = triplet, m = multiplet.

ESI spectra were recorded on a Micromass Quattro API micro (Waters Corporation, Milford, MA, USA) mass spectrometer. Data were processed using a MassLynx System (Waters).

UV/Vis spectra were recorded with a Jasco V-560 spectrophotometer using quartz cuvettes with an optical path length of 1 cm. Fluorescence emission spectra were recorded with a Jasco PP 8350 spectrofluorimeter using quartz cuvettes with an optical path length of 1 cm, using the following parameters: λ_exc_ = 425 nm, λ_emiss_ = 435 – 750 nm.

Fluorescence lifetimes were recorded with Spex Fluorolog-2 (mod. F-111) fluorimeter equipped with a TCSPC Triple Illuminator. The samples were irradiated by a pulsed diode excitation source Nanoled at 455 nm. The kinetic was monitored at 536 nm and each solution itself was used to register the prompt at 455 nm. The system allowed measurement of fluorescence lifetimes from 200 ps. The multiexponential fit of the fluorescence decay was obtained using equation 1:

*I*(*t*) = Σα_i_exp(^-t/τi^) (1)

Photolysis experiments were performed by irradiating the samples in solution with a blue laser (λ_exc_ = 405 nm, 100 mW for **HA-NOPD1** and 20 mW for **HA-NOPD2**) using thermostated quartz cuvettes with an optical path length of 1 cm under continuous stirring.

Direct monitoring of NO release in solution was performed by amperometric detection with a World Precision Instrument, ISO-NO meter, equipped with a data acquisition system, and based on direct amperometric detection of NO with a short response time (< 5 s) and sensitivity range of 1 nM–20 mM. The analog signal was digitalized with a four-channel recording system and transferred to a computer. The sensor was accurately calibrated by mixing standard solutions of NaNO_2_ with 0.1 M H_2_SO_4_ and 0.1 M KI according to the reaction:

4H^+^ + 2I^–^ + 2NO_2_^–^ → 2H_2_O + 2NO + I_2_

Irradiation was performed in a thermostated quartz cell (1 cm path length, 3 mL capacity, 25 1C) by using the above-mentioned blue laser (λ_exc_ = 405 nm, 100 mW for **HA-NOPD1** and 20 mW for **HA-NOPD2**). NO measurements were carried out under stirring with the electrode positioned outside the light path to avoid NO signal artifacts due to photoelectric interference on the ISO-NO electrode.

FT-IR analyses were performed using a Perkin-Elmer FTIR System 2000 (Waltham, MA, USA) equipped with an ATR accessory. Spectra were recorded in the range of 4000–450 cm⁻¹ with a total of 20 scans to enhance the signal-to-noise ratio.

**Synthesis**

The HA derivatives **HA-NOPD1**, **HA-NOPD2** and **HA-PD2** have been synthesized following Scheme 1.

*Synthesis of 4-((4-Nitro-3-trifluoromethylphenyl)amino)butanoic acid (NOPD1).* NOPD1 was synthesized as reported by Parisi and coworkers^[1]^.

*Synthesis of 2,5-dioxopyrrolidin-1-yl 6-((7-nitrobenzo[c][1,2,5]oxadiazol-4-yl)amino)hexanoate* *(PD2).* PD2 was synthesized as reported by Greco and coworkers^[2]^.

*Synthesis of 2,5-dioxopyrrolidin-1-yl-6-((7-nitrobenzo[c][1,2,5]oxadiazol-4-yl) (nitroso)amino)hexanoate (NOPD2).* NOPD2 was prepared according to literature^[3]^ but with minor modifications. To a solution of PD2 (0.256 mmol, 100 mg) in a THF/CH_3_COOH mixture (1/1, 10 mL) cooled at 0 °C with an ice bath, sodium nitrite (1.024 mmol, 71 mg) was added in one portion. The reaction mixture was kept stirring for 30 minutes at 0°C and for 12 hours at room temperature and then diluted with 30 mL of DCM. The resulting solution was washed with water (2 x 20 mL); the organic layer was dried and concentrated to dryness. Purification of the residue by flash chromatography, using petroleum ether/EtOAc (60/40, v/v) as the eluent, gave NOPD2 as a yellow solid (76 mg, 71%). ^1^H NMR (500 MHz, CDCl_3_) δ = 8.61 (d, J = 8.1 Hz, 1H), 7.82 (d, J = 8.2 Hz, 1H), 4.46 – 4.35 (m, 2H), 2.84 – 2.81 (m, 4H), 2.62 – 2.51 (m, 2H), 1.89 – 1.83 (m, 2H), 1.66 – 1.59 (m, 4H). ^13^C NMR (126 MHz, CDCl_3_) δ = 169.5, 169.3, 144.7, 144.0, 137.3, 131.9, 114.6, 60.6, 44.1, 30.7, 26.5, 25.8, 24.0. ESI-MS [M-H]-: m/z 419.5.

*Synthesis of HA-NH_2_*. HA-NH_2_ was prepared as described by Thierry et al.^[4]^ but using 1/0.6 as the ratio of HA/EDA∙HCl equivalents. Specifically, HA (0.374 mmol, 150 mg) and EDC∙HCl (0.486 mmol, 93 mg) were dissolved in 10 mL MES buffer (0.1 M, pH = 5.0). EDA∙2HCl (0.224 mmol, 30 mg) was added and the resulting solution was kept overnight at room temperature. The reaction mixture was purified by dialysis against distilled water using a Spectra/Por^®^ regenerated cellulose membrane (Spectrum, Breda, The Netherlands, MWCO = 3.5 KDa) and recovered by freeze-drying to produce HA-NH_2_ (140 mg, 92%). As reported by Thierry et al.^[4]^, ^1^H NMR spectrum in D_2_O confirmed the presence of the ethylenediamine groups. The signal at δ = 3.05 ppm attributable to the ethylene protons of the ethylenediamine moieties and, the singlet of the acetamide methyl protons resonance at δ = 1.89 ppm, were used to calculate the degree of functionalization of HA-NH_2_ which resulted in 0.3 mol ethylene diamine/disaccharide unit.

*Synthesis of* ***HA-NOPD1*.** To a solution of HA-NH_2_ (0.07 mmol, 30 mg) in phosphate buffer (2 mL, 3 mM, pH 6.5) was added a solution of NOPD1 (0.182 mmol, 53 mg) in DMF (2 mL). The resulting mixture was stirred at room temperature for 24 hours. It was purified by dialysis, first against 50% acetone/distilled water for 6 hours in order to remove unreacted NOPD1, then against distilled water for 24 hours, using a Spectra/Por^®^ regenerated cellulose membrane (Spectrum, Breda, The Netherlands, MWCO = 3.5 KDa). Recovery by freeze-drying gave **HA-NOPD1** (5 mg, 16%) as a yellow solid. ^1^H NMR spectrum in D_2_O confirmed the presence of the NOPD1 moiety (Figure S1). The multiplets at 1.92, 2.32, and 3.31 ppm are associated with the aliphatic chain linked to the aromatic ring of the NOPD1 moiety, while the signals at 6.86, 7.10, and 8.14 ppm correspond to its aromatic portion. FTIR analysis also demonstrated the successful anchoring of NOPD1 at the polymer. Spectrum c in Figure S2 shows bands at 1723 cm⁻¹ and 3383 cm⁻¹, which can be attributed to the C=O stretching and O-H stretching vibrations of the carboxylic groups, respectively. In contrast, the IR spectrum of **HA-NOPD1** (spectrum d in Figure S2) shows a band shifted to 1611 cm⁻¹, which can be attributed to the C=O stretching vibration of the amide group suggesting the formation of the amide bond. Additionally, the band at 3383 cm⁻¹ has disappeared, and this region is now dominated exclusively by the O-H stretching vibration of the hydroxyl groups of the HA. The –N=O stretching vibration at 1550 cm⁻¹ is visible in free NOPD1 as well as in **HA-NOPD1**.

*Synthesis of* ***HA-NOPD2***. To a solution of HA-NH_2_ (0.07 mmol, 30 mg) in phosphate buffer (2 mL, 3 mM, pH 6.5) was added a solution of NOPD2 (0.182 mmol, 77 mg) in DMF (2 mL). The resulting mixture was stirred at room temperature for 24 hours. It was purified by dialysis, first against 50% acetone/distilled water for 6 hours in order to remove unreacted NOPD2, then against distilled water for 24 hours, using a Spectra/Por^®^ regenerated cellulose membrane (Spectrum, Breda, The Netherlands, MWCO = 3.5 KDa). Recovery by freeze-drying gave **HA-NOPD2** (20 mg, 67%) as a yellow solid. ^1^H NMR spectrum in DMSO confirmed the presence of the NOPD2 moiety (Figure S3) The choice of solvent was dictated by solubility issues at the desired concentration, as well as instability concerns in D_2_O. The signals at 1.53–1.59, 2.62, and 4.27 ppm are associated with the aliphatic chain linked to the aromatic ring of the NOPD2 moiety, while the doublets at 7.87 and 8.78 ppm correspond to its aromatic portion. FTIR analysis was also performed to confirm the successful anchoring of NOPD2 to the polymer. Spectrum d in Figure S4 shows a band at 1628 cm⁻¹, which can be attributed to the C=O stretching vibration of the amide group, suggesting the formation of the amide bond. Additionally, the broad band at 3291 cm⁻¹ arises from the O-H stretching vibration of the hydroxyl groups in HA, and the –N=O stretching vibration at 1537 cm⁻¹ is visible in both free NOPD2 (spectrum c in Figure S4) and **HA-NOPD2** (spectrum d in Figure S4).

*Synthesis of* ***HA-PD2.*** To a solution of HA-NH_2_ (0.07 mmol, 30 mg) in phosphate buffer (2 mL, 3 mM, pH 6.5) was added a solution of PD2 (0.182 mmol, 71 mg) in DMF (2 mL). The resulting mixture was stirred at room temperature for 24 hours. It was purified by dialysis, first against 50% acetone/distilled water for 6 hours in order to remove unreacted PD2, then against distilled water for 24 hours, using a Spectra/Por^®^ regenerated cellulose membrane (Spectrum, Breda, The Netherlands, MWCO = 3.5 KDa). Recovery by freeze-drying gave HA- PD2 (18 mg, 60%). ^1^H NMR spectrum in D_2_O confirmed the presence of the PD2 moiety (Figure S5). The ^1^H-NMR spectrum in D_2_O confirmed the presence of the PD2 moiety. Specifically, the signals at 6.41 and 8.55 ppm arise from the aromatic portion of PD2, while the signals at 1.1 and 2.1 ppm correspond to part of the aliphatic chain. The remaining aliphatic signals are masked by overlapping signals of the HA. FTIR analysis was also performed to confirm the successful anchoring of PD2 to the polymer. Spectrum d in Figure S6 shows a band at 1628 cm⁻¹, which can be attributed to the C=O stretching vibration of the amide group, suggesting the formation of the amide bond. Moreover, the band at 3313 cm⁻¹, visible in spectrum c of Figure S6 and attributed to the O-H stretching of the carboxylic group, has disappeared in **HA-PD2** (spectrum d of Figure S6). This region is now dominated exclusively by the O-H stretching vibration of the hydroxyl groups in HA.

*Degree of substitution*. The NOPD1, NOPD2 and PD2 content was determined by UV absorbance spectroscopy analysis of **HA-NOPD1**, **HA-NOPD2** and **HA-PD2** solutions in MeOH/Water 50/50, using NOPD1 (λ = 398 nm), NOPD2 (λ = 384 nm) and PD2 (λ = 384 nm) as references, respectively. UV/Vis spectra absorption was recorded in air-equilibrated solutions using quartz cells with a 1 cm path length and a 3 mL capacity.


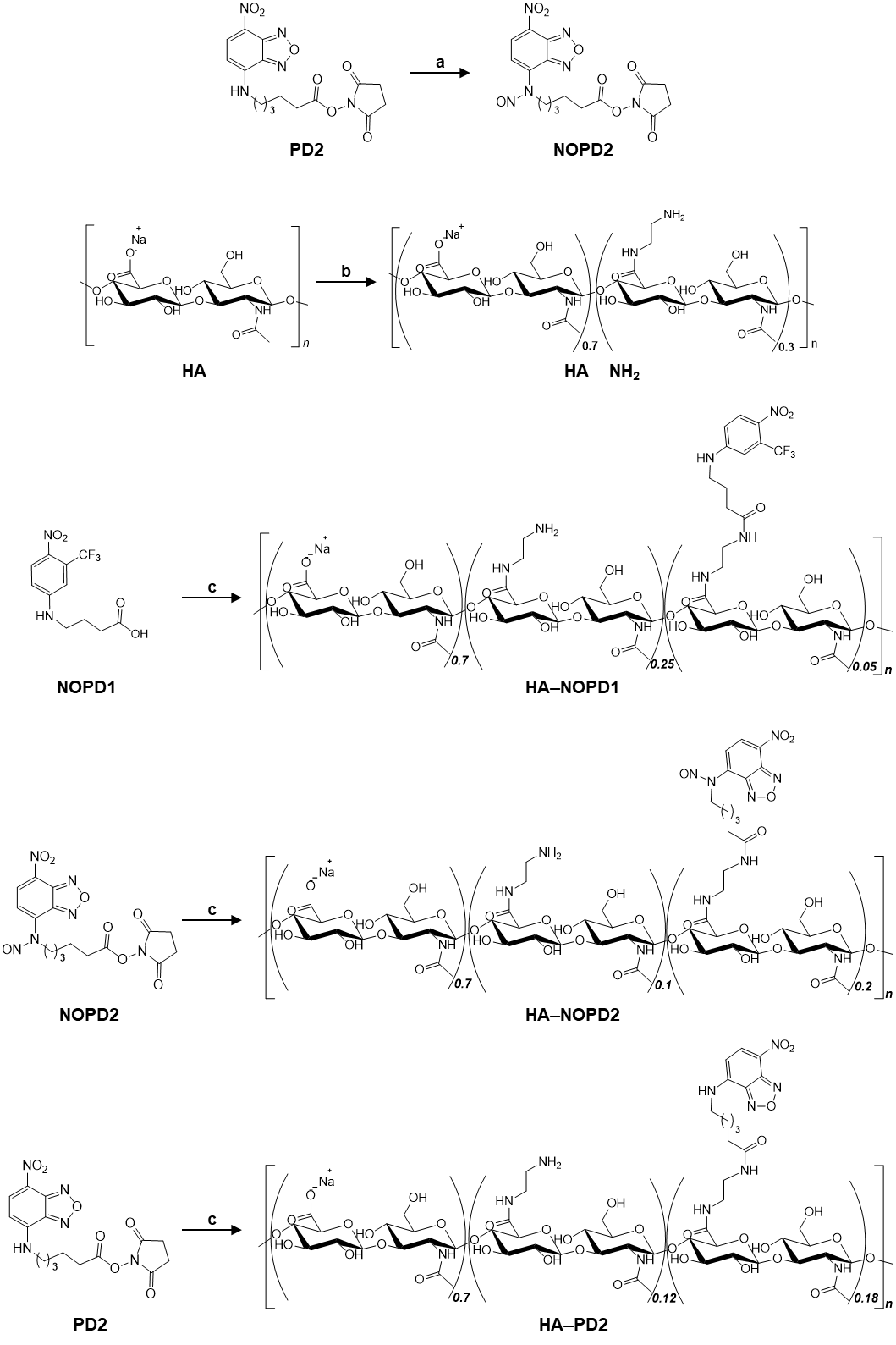


**Scheme 1.** a) NaNO_2_, THF/CH_3_COOH (1/1 v/v), 12 h, 0°C → r.t.; b) EDC · HCl, EDA· 2HCl, MES buffer, 12 h, r.t.; c) HA-NH2, PBS (3 mM, pH 6.5)/DMF (1/1 v/v), 24 h, r.t.

**Experimental Procedures**

*Photodecomposition quantum yields*. Photodecomposition quantum yield (Φ_NO_) was determined at λ_exc_ = 405 nm within the 20% transformation of the conjugates by using equation 2.

$\Phi_{NO}=\frac{\left[ C \right]\times V}{t\times(1-{10}^{-A})\times I}$ (2)

where [C] is the concentration of phototransformed **HA-NOPD1** or **HA-NOPD2**, V is the volume of the irradiated sample, t is the irradiation time, A is the absorbance of the sample at the excitation wavelength and I the intensity of the excitation light. the concentration of phototransformed **HA-NOPD1** or **HA-NOPD2** were determined spectrophotometrically by considering the absorption changes at 380 nm and Δε_380_ = 10000 for **HA-NOPD1** and Δε_380_ = 7700 for **HA-NOPD2**.

*Development of oil-in-water (O/A) microemulsions*. For the preparation of oil-in-water (O/A) microemulsions, a precise amount of water was slowly added to the oil phase (oil plus surfactant) under continuous magnetic stirring. The microemulsions’ formation area was determined by constructing a pseudo-ternary phase diagram. A titration method was used to construct the phase diagrams, using Kolliphor^®^ HS 15 as the surfactant, Labrafac^TM^ Lipophile WL1349 as the oil phase, and water. Initially, various mixtures of Kolliphor^®^ HS 15 and Labrafac^®^ Lipophile WL1349 were added to different vials in ratios ranging from 9:1 to 1:9 and placed under magnetic stirring for 1 hour. Finally, the aqueous phase was slowly added with a graduated syringe, under vigorous agitation, until the oil and surfactant mixture became clouded. Approximately 10 points were required to determine each region of the pseudo-ternary diagram. No attempts were made to identify in detail the other regions of the phase diagram; therefore, they were described only in terms of their macroscopic appearance. To visually represent the distinct regions of the Kolliphor^®^ HS 15 – water –Labrafac^TM^ Lipophile WL1349 system, a pseudo-ternary phase diagram was constructed using OriginPro software.

Once the region of microemulsion (ME) existence was defined, a series of tests were conducted to determine the most suitable ratios of surfactant, oil phase, and water to form MEs with extended colloidal stability (Table S1). Formulations with different component ratios were prepared at room temperature and characterized after 24 hours, allowing equilibrium to be reached. Samples exhibiting phase separation, flocculation, or excessive turbidity after 24 hours were discarded.

By definition, microemulsions (MEs) are transparent (size <100 nm) and isotropic systems. Therefore, all formulations that exhibited a milky appearance or macroscopic phase separation after 24 hours of stabilization were excluded. Confirmation of system formation was obtained by Dynamic Light Scattering (DLS), evaluating the size (D_H_) and polydispersity index (PDI) of the formulated MEs using a Zetasizer^®^ Nano-ZS ZEN 3600, (Malvern instruments, Worcestershire, UK) equipped with a red laser light beam (λ=632.8 nm). Only formulations with a size <100 nm and PDI < 0.2 were classified as microemulsions. Finally, the short-term stability of the remaining formulations was assessed by storing the microemulsions tightly sealed in their containers under two different conditions (37°C and at room temperature) for at least one week. This preliminary screening led to the identification of a formulation (composed of 10% Kolliphor^®^ HS 15, 5% Labrafac^TM^ Lipophile WL1349, and 85% water) that met the selection criteria.

*Preparation and characterization of microemulsions with HA-NOPDs.* The ME containing **HA-NOPD1** (ME_**HA-NOPD1**) was prepared by adding 0.5 mg of **HA-NOPD1** to 2 mL of the selected ME (10% Kolliphor^®^ HS 15.5% Labrafac^TM^ Lipophile WL1349, and 85% water) under magnetic stirring at 900 RPM (final concentration: 0.25 mg/mL). ME_**HA-NOPD1** was stirred for 3 h and let rest for an additional 30 min. ME containing **HA-NOPD2** (ME_**HA-NOPD2**) were prepared in the same way, by adding 0.5 mg of **HA-NOPD2** to 2 mL of the selected ME. As control an empty ME and a ME loaded with PD2 (ME_**HA-PD2**) were prepared.

The average hydrodynamic diameter (DH), polydispersity index (PI) and zeta potential (ζ) were determined on a Zetasizer® Nano-ZS (Malvern, UK). The pH of each formulation was assessed using a Crison basic 20 pH meter equipped with an electrode 50 10T (Crison, Spain). The conductivity of the MEs was determined using a Crison model GLP 31 laboratory conductivity meter (Crison, Spain).

The morphology of the MEs droplets was evaluated by TEM with a FEI Tecnai G12 microscope equipped with a FEI Eagle 4K CCD camera (Eindhoven, The Netherlands) operating at an accelerating voltage of 120 kV. Sample analysis was performed upon air drying (~ 1 hour) of 10 µL of ME-**HA-NOPD2** mounted on 200 mesh copper grids. Before analysis, the MEs were stained with phosphotungstic acid (2%, w/v).

The colloidal stability of the ME was monitored by spectroscopic and colloidal analyses for up to 3 days under in the dark at room temperature. At predetermined time intervals, samples were withdrawn and analysed, determining particle size, zeta potential, (as described in the previous section) and to describe the appearance of the suspension. The degradation of the loaded MEs was evaluated by monitoring the changes in the absorption spectrum in the same time intervals as reported above.

*Structural Arrangement of the HA derivatives in the MEs.* The arrangement of the bioconjugate within the ME was analyzed by Förster resonance energy transfer analysis (FRET). For this purpose, FRET-ME were prepared as mentioned earlier by adding 1.25 mg of DiI to the oil/surfactant mixture and using the **HA-PD2** derivative (fluorescent). ME were also prepared with **HA-PD2** and with only DiI as a control. The fluorescence emission spectra of the FRET-ME were collected at the excitation wavelength of **HA-PD2** (λ_exc_ = 470 nm) with a fluorescence spectrometer (RF-6000, Shimadzu Corporation, Tokyo, Japan).

*Cell culture*. Human epidermal keratinocytes (HaCaT cells) were purchased from CEINGE Biotecnologie Avanzate – Franco Salvatore Cell Culture Facility (Napoli, Italy) and grown in DMEM supplemented with 10% FBS, 100 U/mL penicillin, 100 μg/mL streptomycin, and 2 mM L-Glutamine. Cells were sub-cultured every three days at a 1:5 ratio, maintained at 37 °C in a humidified atmosphere containing 5% CO_2_, and tested for mycoplasma contamination before starting the experiments. Both HA and **HA-NOPD1** derivatives were dissolved in PBS containing 5% DMSO to prepare stock solutions at 2 mg/mL concentration. These solutions were sterilized through a 0.22 μm filter and freshly diluted in cell culture medium to the desired concentrations before each experiment. The **HA-NOPD1** stock and working solutions were handled under dark conditions to avoid premature NO release.

*Cell viability assay*. The cytotoxicity of both HA and the **HA-NOPD1** derivative towards HaCaT cells was assessed using the standard colorimetric MTT assay^[5]^. Briefly, HaCaT cells were seeded in 96-well plates at a density of 25,000 cells/well and incubated at 37 °C for 24 h. Before irradiation with blue light (21.9 mW/cm^2^ at 420 nm) for 15 minutes, the cell culture medium was replaced with phenol red-free medium containing increasing concentrations (50–600 µg/mL) of either HA or **HA-NOPD1**. Cells were then incubated at 37 °C for further 24 h. As a control, the same treatments were conducted under dark conditions. At the end of the treatments, 20 μL of CellTiter 96^®^ AQueous One Solution Reagent were added to each well and the plates were then maintained at 37 °C for 4 h. Finally, the absorbance of the samples at 490 nm was recorded using the GloMax^®^ Discover Microplate Reader (Promega, Madison, WI, USA) and the percentage (%) of cell survival, for each condition, was determined using equation 3.

$\% of cell survival= \frac{Abs - \mathrm{Abs}_{0}}{\mathrm{Abs}_{\mathrm{Control}}- \mathrm{Abs}_{0}} x 100$ (3)

where Abs is the absorbance of the sample, Abs_0_ is the absorbance of the background signal, and Abs_Control_ is the absorbance of the control sample (cells treated with the vehicle).

Representative light microscope images of HaCaT cells treated for 24 h were collected under dark conditions or after 15 minutes of irradiation with blue light (21.9 mW/cm^2^ at 420 nm) using a Mateo TL inverted microscope (Leica Microsystems, Wetzlar, Germany) at x10 magnification.

*Wound healing assay.* HaCaT cells (40,000) were seeded in each well of the Ibidi culture inserts and incubated at 37 °C until 100% confluence was reached. Cell proliferation was inhibited by exposing cells to Mitomycin-C (5 μg/mL) for 4 h. Afterwards, the inserts were removed with sterile tweezers to create a cell-free area and 1 ml of phenol red-free medium containing 200 µg/mL HA or **HA-NOPD1** was added. Cells were then irradiated with blue light (21.9 mW/cm^2^ at 420 nm) for 15 min and allowed to migrate into the cell-free zone for 24 h. Cell images at 0- and 24-h post-scratch were captured with a Mateo TL inverted microscope (Leica Microsystems, Wetzlar, Germany) at x10 magnification, and quantified using Fiji software. The percentage of closing area was calculated according to equation 4:

$\text{\% of closing area= }\frac{\text{Wound area}_{\text{T0}}\text{ - }\text{Wound area}_{\text{T24}}}{\text{Wound area}_{\text{T0}}}\text{ x 100}$ (4)

where Wound area_T0_ represents the area of the cell-free gap at time 0 (immediately after the insert removal) and Wound area_T24_ is the area of the wound 24 h post-scratch. As a control, the same treatments were also conducted under dark conditions.

*Permeation studies*. Drug transport through a syntenic membrane (Strat-M^®^ 25 mm) and porcine ear skin was assessed using jacked Franz-type diffusion cells (diffusion area 1 cm^2^). Porcine ear skin was obtained from a porcine weighing about 100 kg, slaughtered on the day of the experiment. After removal, the tissue was stored in phosphate buffer saline (PBS, 120 mM sodium chloride, 2.7 mM potassium chloride, 10 mM sodium phosphate monobasic) at 4 °C and used within 2 h. The skin was first cleaned with distilled water, shaved to remove all the surface hairs, and then separated from the underlying tissue by surgical excision. The porcine ear skin sample was mounted between the donor and acceptor compartments with the stratum corneum facing the tested formulation. For the permeation experiments, 200 μL of the tested ME_**HA-NOPD2** was spread over the membrane on the donor compartment, while the acceptor compartment was filled with 8 mL of PBS at pH 7.4, containing 10% v/v polysorbate 80. The donor compartment was sealed to prevent sample evaporation. The experiment was conducted in the dark at 35 ± 1 °C under gentle agitation. The experiment on porcine ear skin was performed under environmental light (to assess spontaneous NO release) and in the dark (to subsequently assess NO release controlled by blue light). At predetermined times (1, 2, 4, 6, 24 h), 300 μL of acceptor solution was withdrawn and replaced with an equivalent volume of fresh medium. To quantify the **HA-NOPD2** permeated into the acceptor at different times, the samples were photodegraded under environmental light for 2 h, and fluorescence intensity was analyzed (λ_exc_ 450 nm / λ_emiss_ 537 nm). The amounts of permeated **HA-NOPD2** were then calculated using a calibration curve in the concentration range 0.068-17.2 mg/mL. Results are reported as μg/cm2 of **HA-NOPD2** permeated ± SD of three different experiments.

*Nitrite assay*. NO released during the permeation experiment through the porcine ear skin conducted under the environment light was quantified by a Griess reaction-based nitrite assay according to the manufacturer’s protocol (Thermo Fisher Scientific, Waltham, MA, USA). Briefly, 100 μL of the acceptor solution was mixed with 10 μL of Griess Reagent I, 10 μL of Griess Reagent II, and 80 μL of the buffer solution from the kit. The samples were then incubated for 10 minutes under agitation, and absorbance was measured at 540 nm using the Promega GloMax^®^ plate reader. As a control, the same assay was performed on porcine ear skin treated with 200 μL of PBS (CTR). The amount of nitrite produced was calculated using a calibration curve prepared with the sodium nitrite standard from the kit, covering a concentration range of 5-25 nmol/mL.

*Skin distribution of MEs*. The distribution of the **HA-NOPD2** in the skin sample after permeation experiments was assessed by Laser Scanning Confocal microscopy. At the end of the permeation experiment (4 and 24 h), the skin samples were carefully recovered, washed with distilled water, and gently dried with a cotton swab before being fixed in 4% paraformaldehyde for 48 h. Sections (20 μm) were obtained from frozen tissue blocks in Optimal Cutting Temperature (OCT, Sakura Finetek) using a Leica CM1850 cryostat. Cutting was performed from the dermis side toward the epidermis to prevent the dislocation of microemulsions from the skin surface of the section. Three skin sections were taken from each treated area. A control sample prepared by replacing the MEs with 200 μL of PBS over the donor compartment was run. The samples sections were then stored on polylysine slides at 20 °C and were analyzed within 24 h on a confocal microscope (Zeiss LSM 700 Confocal Laser Scanning Microscope) equipped with Argon (488 nm) and HeNe (561, 639 nm) lasers and a 40X/1.2 NA objective. To ensure a proper visual comparison, we kept the exposure time constant for all the samples examined, including controls.

*Statistical Analysis*. Statistical analyses were undertaken using GraphPad Prism^®^, version 10 (GraphPad Software, La Jolla California USA) and OriginPro, Version Number 2022 (OriginLab Corporation, Northampton, MA, USA). All experiments were performed in triplicate and the results were expressed as the average ± standard deviation (SD). One-way analysis of variance (ANOVA) was performed for comparisons among multiple groups. P values < 0.05 were considered statistically significant.

**References**

[1] C. Parisi, M. Failla, A. Fraix, L. Menilli, F. Moret, E. Reddi, B. Rolando, F. Spyrakis, L. Lazzarato, R. Fruttero, A. Gasco, S. Sortino, *Chemical Science* **2021**, 12, 4740.

[2] G. Greco, N. D’Antona, G. Gambera, G. Nicolosi, *Synlett* **2014**, 25, 2111.

[3] a)C. Parisi, M. Failla, A. Fraix, B. Rolando, E. Gianquinto, F. Spyrakis, E. Gazzano, C. Riganti, L. Lazzarato, R. Fruttero, A. Gasco, S. Sortino, *Chemistry – A European Journal* **2019**, 25, 11080; b)C. Parisi, M. Seggio, A. Fraix, S. Sortino, *ChemPhotoChem* **2020**, 4, 742.

[4] B. Thierry, P. Kujawa, C. Tkaczyk, F. M. Winnik, L. Bilodeau, M. Tabrizian, *Journal of the American Chemical Society* **2005**, 127, 1626.

[5] T. Mosmann, *J Immunol Methods* **1983**, 65, 55.

**Table S1.** Properties of the 29 different MEs prepared by testing different ratios between surfactant, oil phase and water.

| **Sample N.** | **Oily phase (%)** | **Surfactant (%)** | **Water phase (%)** | **Appearance** | |
| --- | --- | --- | --- | --- | --- |
|  | *Labrafac^TM^ WL 1349* | *Kolliphor® HS 15* | *Water* | *Size (nm)* | *PDI* |
| 1 | 2.5 | 5 | 92.5 | Cloudy | |
| 2 | 2.5 | 10 | 87.5 | 23.23 | 0.1 |
| 3 | 2.5 | 15 | 82.5 | 20.64 | 0.2 |
| 4 | 2.5 | 20 | 77.5 | 20.52 | 0.3 |
| 5 | 5 | 5 | 90 | Cloudy | |
| 6 | 5 | 10 | 85 | 37.69 | 0.2 |
| 7 | 5 | 15 | 80 | 29.14 | 0.2 |
| 8 | 5 | 20 | 75 | 29.67 | 0.3 |
| 9 | 6.25 | 6.25 | 87.5 | Cloudy | |
| 10 | 5.9 | 5.9 | 88.2 | Cloudy | |
| 11 | 5.6 | 5.6 | 88.9 | Cloudy | |
| 12 | 5.3 | 5.3 | 89.5 | Cloudy | |
| 13 | 4.8 | 4.8 | 90.5 | Cloudy | |
| 14 | 4.5 | 4.5 | 90.9 | Cloudy | |
| 15 | 4.3 | 4.3 | 91.3 | Cloudy | |
| 16 | 4.2 | 4.2 | 91.7 | Cloudy | |
| 17 | 7.1 | 7.1 | 85.7 | Cloudy | |
| 18 | 10 | 5 | 85 | Cloudy | |
| 19 | 10 | 10 | 80 | Cloudy | |
| 20 | 10 | 15 | 75 | Cloudy | |
| 21 | 10 | 20 | 70 | 74.52 | 0.4 |
| 22 | 10 | 25 | 65 | 60.20 | 0.5 |
| 23 | 15 | 15 | 70 | Cloudy | |
| 24 | 15 | 20 | 65 | Cloudy | |
| 25 | 15 | 25 | 60 | 268.3 | 0.9 |
| 26 | 15 | 30 | 55 | Cloudy | |
| 27 | 20 | 20 | 60 | 569.5 | 0.4 |
| 28 | 20 | 25 | 55 | Cloudy | |
| 29 | 20 | 30 | 50 | Cloudy | |

**Table S2.** Properties of the MEs loaded with the **HA-PD2** and DiI.

|  | D_H_ (nm) | | | PDI | ζ (mV) | | pH | | Conductivity (μS/cm) | |
| --- | --- | --- | --- | --- | --- | --- | --- | --- | --- | --- |
| ME_HA-PD2/Dil | | 38 ± 1 | 0.2 | | | -0.9 ± 0.1 | | 5.9 ± 0.1 | | 166 ± 2.8 |
| ME_DiI | | 39 ± 1 | 0.2 | | | -0.9 ± 0.1 | | 5.9 ± 0.1 | | 160 ± 2.8 |


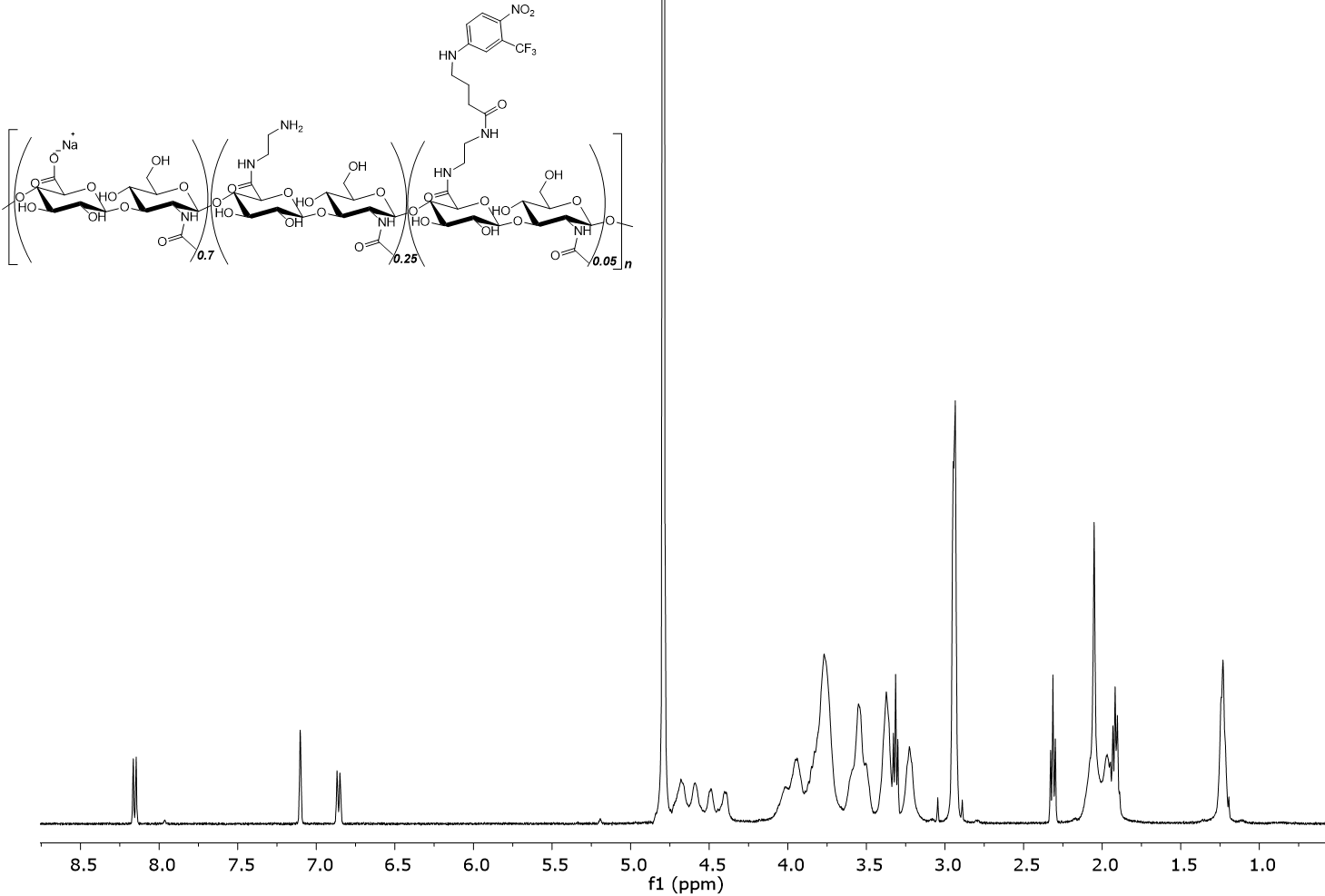


**Figure S1.** ^1^H-NMR of **HA-NOPD1** in D_2_O.

**Figure S2.** FTIR spectra of HA (a), HA-NH_2_ (b), NOPD1 (c) and **HA-NOPD1** (d).


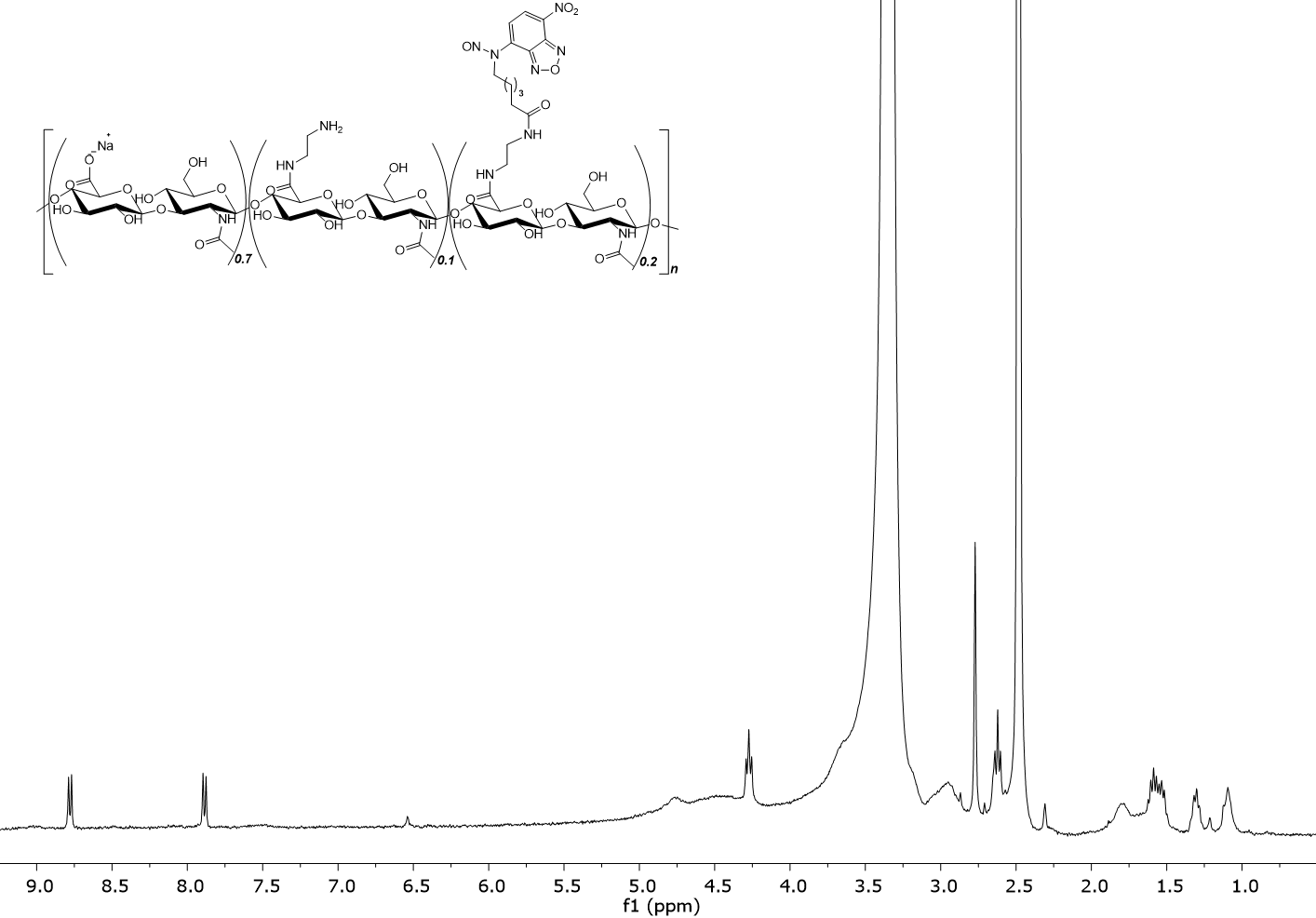


**Figure S3.** ^1^H-NMR of **HA-NOPD2** in DMSO.

**Figure S4.** FTIR spectra of HA (a), HA-NH2 (b), NOPD2 (c) and **HA-NOPD2** (d).


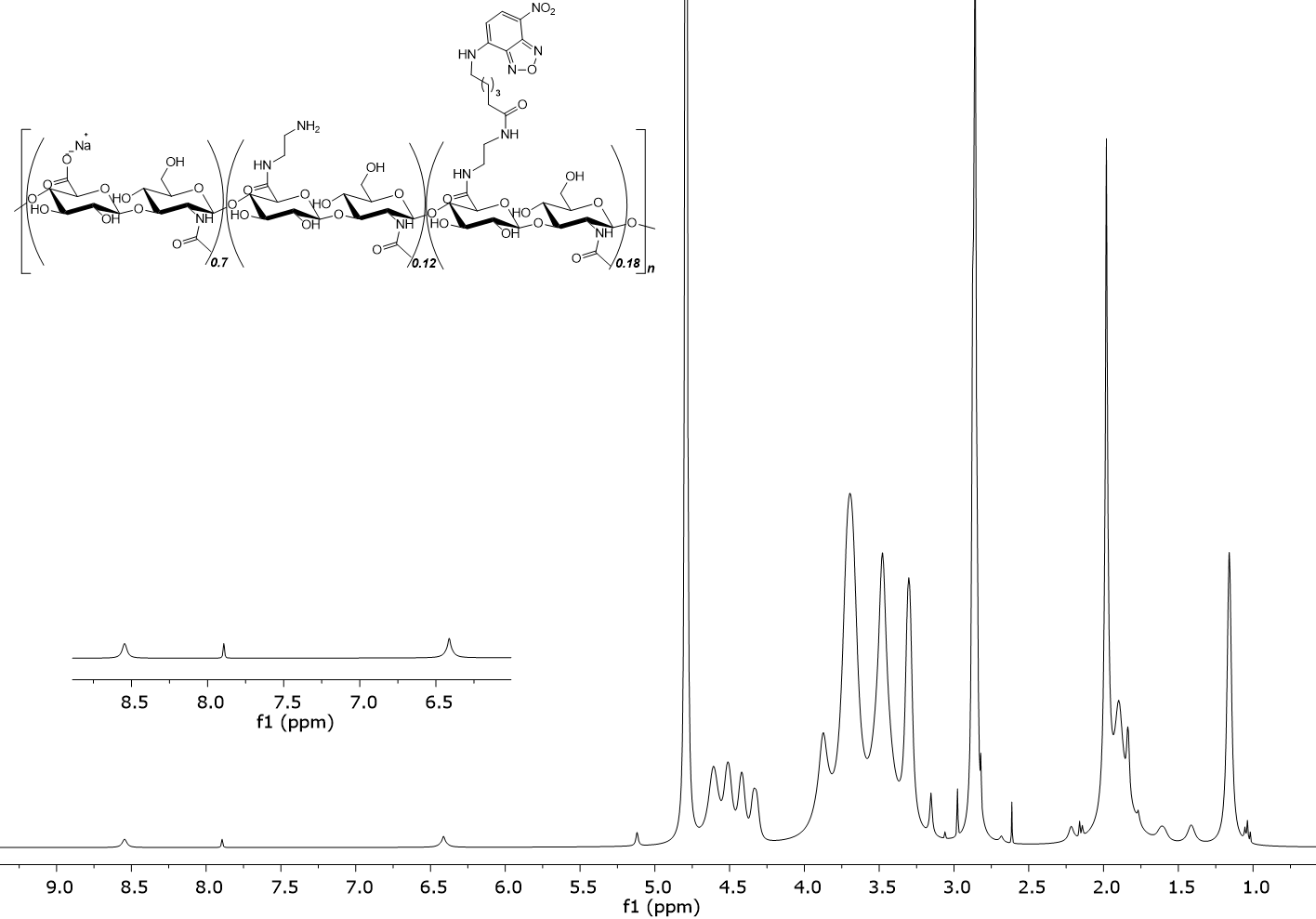


**Figure S5.** ^1^H-NMR of **HA-PD2** in D_2_O.

**Figure S6.** FTIR spectra of HA (a), HA-NH_2_ (b), PD2 (c) and **HA-PD2** (d).

**Figure S7.** Fluorescence decay and the related fitting of **HA-NOPD2** after irradiation 13 min of irradiation at λ_ac_ = 405 nm, recorded at λ_exc_ = 455 nm and λ_em_ = 550 nm. H_2_O: MeOH (1:1 v/v), T = 25 °C.

**
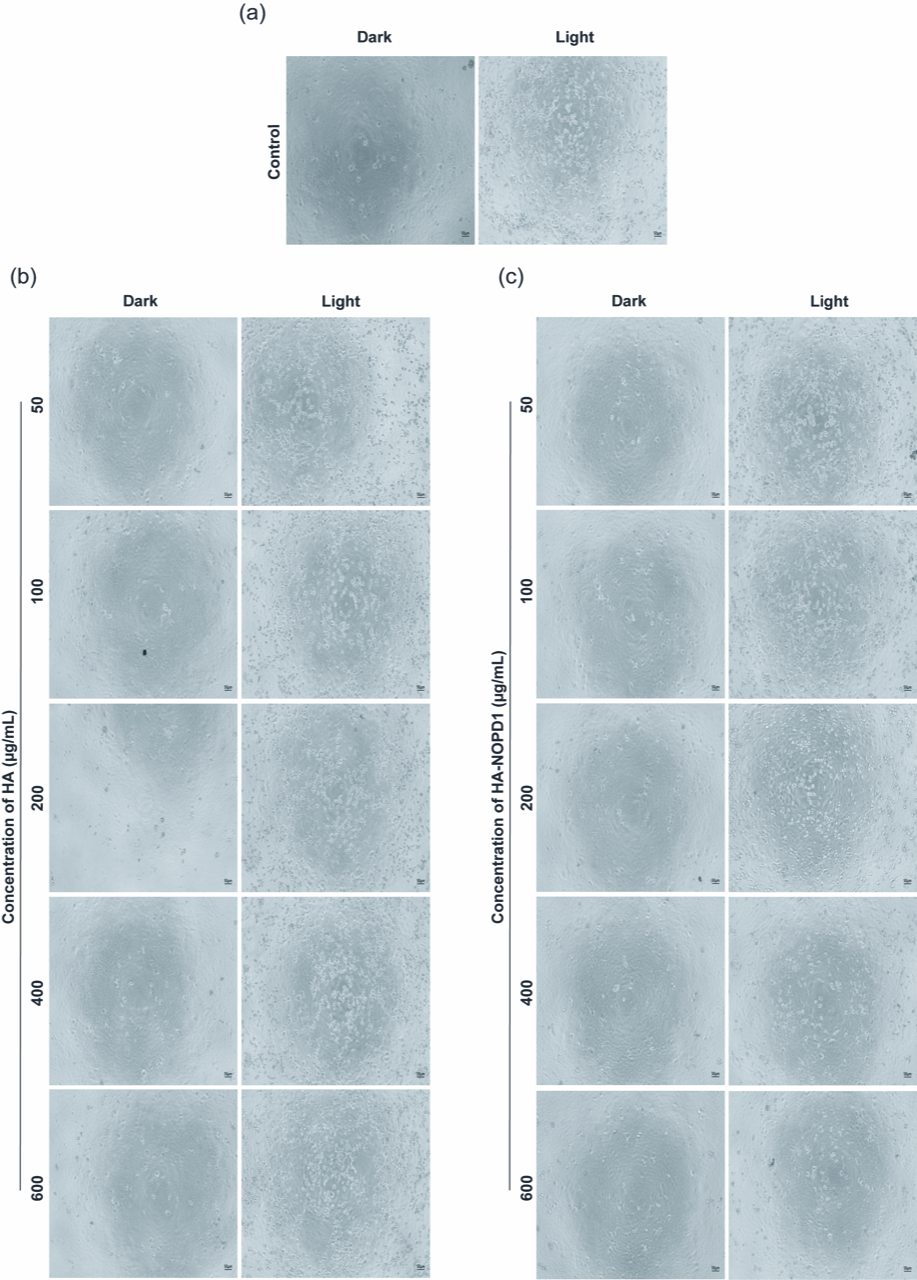
**

**Figure S8.** Representative light microscope images of HaCaT cells treated for 24 h with either (a) vehicle or increasing concentrations of (b) HA or (c) **HA-NOPD1**, under dark conditions or after 15 minutes of irradiation with blue light (21.9 mW/cm^2^ at 420 nm). Scale bar: 50 µm. The experiments were conducted in two independent biological replicates, each comprising three technical replicates.

**Figure S9.** Absorption spectrum (solid line) and emission at λ_ex_ = 470 nm (dashed line) of the MEs containing **HA-PD2** [25 μg/mL] and DiI [25 μg/mL] individually.
